# Preliminary feasibility and clinical utility of the Unified Protocol for the transdiagnostic treatment of emotional disorders in people with long COVID-19 condition: A single case pilot study

**DOI:** 10.1371/journal.pone.0329595

**Published:** 2025-08-08

**Authors:** Verónica Martínez-Borba, Óscar Peris-Baquero, Iván Prieto-Rollán, Jorge Osma, Esther del Corral-Beamonte

**Affiliations:** 1 Institute for Health Research Aragón (IIS Aragón), Zaragoza, Spain; 2 Department of Psychology and Sociology, University of Zaragoza, Teruel, Spain; 3 Villanova Royo Hospital, Zaragoza, Spain; Hochschule Niederrhein—Campus Mönchengladbach: Hochschule Niederrhein—Campus Monchengladbach, GERMANY

## Abstract

**Purpose:**

The implementation of psychological interventions in long COVID-19 patients is still very limited. This study aims to analyze the feasibility and preliminary utility of the Unified Protocol (UP), for the psychological treatment of emotional disorders in long COVID-19 patients.

**Methods:**

12 long COVID-19 patients (mean age = 47.92; *SD* = 13.18) presenting emotional disorders/symptoms received the UP through 8 online sessions.

**Results:**

All participants completed the UP psychological program, attending all eight sessions and the 6-month follow-up appointment. However, adherence to the assessment protocol was lower, with a 33% dropout rate at the 6-month follow-up. High satisfaction was reported with both the UP program (mean = 9.75) and the online format. Satisfaction with individual UP modules ranged from 7.17 to 9 points (from 0 = any satisfaction to 10 = highest satisfaction). Qualitative feedback emphasized the usefulness of the UP program, with some participants suggesting additional contents (i.e., training in assertive communication skills), more personalized modules (i.e., providing just some UP modules) or increasing the number of sessions. In terms of the UP clinical utility, 25 mental clinical diagnoses were established at pre-assessment; 50% of participants no longer met criteria for a mental clinical diagnosis at post-assessment, increasing to 67% at follow-up. Most therapeutic objectives were achieved or initiated over the course of the intervention (78% at post-assessment; 86% at follow-up). At post-assessment significant improvements were observed in anxiety (8 participants), depression (9 participants), emotion dysregulation (4 participants) and quality of life scores (7 participants), although 2 participants did not maintain these gains in emotion regulation and quality of life at follow-up.

**Conclusion:**

The promising results of the UP in terms of high participant satisfaction and clinical improvements in anxiety and depression scores suggest that the UP could be a valuable psychological intervention for individuals with long COVID-19 and comorbid emotional disorders. The modest improvements found in other outcomes highlighted the need to better adapt the psychological interventions for long COVID-19 patients.

## 1. Introduction

According to the World Health Organization (WHO) communicable or infectious diseases, include HIV/AIDS, tuberculosis, malaria, neglected tropical diseases or the recent COVID-19 pandemic [1]. Despite advances in screening programs, medicines, vaccines and health policies [2], infectious diseases remain highly prevalent worldwide, with millions of new cases reported in 2022 [3].

These high prevalence rates impose a significant economic burden, with WHO reporting investments of US $800 million in communicable diseases [2]. Also importantly, all these medical conditions, and specially their chronicity, are related with high emotional burden [4]. Thus, the co-occurrence of medical conditions and emotional disorders (namely mood, anxiety and related disorders) [5–8] is not negligible. The National Institute for Health and Care Excellence (NICE) has established that patients with chronic physical conditions should be assessed for mental health issues and offered the most appropriate psychological treatment, specifically Cognitive Behavioral Treatment (CBT). However, traditional CBT programs present some limitations; for instance, focusing on specific symptoms which may hinder treatment of comorbid disorders and involve high training costs [9].

The Unified Protocol (hereafter, UP) is a transdiagnostic CBT-based psychological intervention which emerged as an alternative to symptoms-specific protocols. Contrary to traditional CBT approaches, the UP has been designed to address psychopathological mechanisms shared by emotional disorders, such as neuroticism, and the associated emotion regulation difficulties, perfectionism, rumination and avoidance, among others [10], which facilitate treating comorbidity and reducing the training needed by professionals [11]. The versatility of the UP allows the number of sessions to be adapted to the context and characteristics of the participants, while maintaining its effectiveness [12]. The UP has demonstrated its efficacy in the treatment of emotional disorders in adults with emotional disorders and comorbid medical conditions such as breast cancer, irritable bowel syndrome, chronic pain, Parkinson’s disease, multiple sclerosis, HIV or infertility [13,14]. However, their clinical utility and feasibility in important infectious diseases like COVID-19 conditions has not yet been analyzed.

COVID-19 conditions are relevant not only for the impact they had in the entire society from the beginning, but also for the negative consequences that are still present in millions of people. From the first COVID-19 pandemic to December 2024, there have been 778 million confirmed cases and 7.1 million deaths worldwide [15]. Of those, 14 million confirmed cases and 122.000 deaths were reported in Spain [15]. While most confirmed cases have recovered, it is also important to note the worrisome proportion of people that still have physical and emotional symptoms as a result of the COVID-19 infection. This is now known as post COVID-19 or long COVID-19 condition. According to the WHO, the long COVID-19 condition affects approximately 10−20% of patients that have not recovered from COVID-19 symptoms, or have even developed new symptoms, three months after being infected [16]. A recent study showed that, in Spain, the prevalence of long COVID-19 symptoms was approximately 41% at 6 months after the initial infection, decreasing to 8.6% at 12 months post-infection [17]. Long COVID-19 patients have to cope not only with chronic physical complaints but also with the emotional burden associated with living with an unknown medical condition [18,19]. Not surprisingly, some reports indicate that anxiety and depressive disorders are present in 21−22% of long COVID-19 patients [17,20]. Recent studies have attempted to explain the connection between the COVID-19 disease and the development of psychological symptoms such as anxiety and depressive symptoms. Some of these ideas are summarized in the study by Fancourt et al. 2023 (see [21]) and in the study by Thye et al. (2022) (see [22]), among others. On the one hand, the authors explain that SARS-CoV-2 infection can trigger neuroinflammation processes that may lead to psychiatric and cognitive symptoms. On the other hand, they also acknowledge that the onset and maintenance of emotional symptoms in COVID-19 patients may also be explained by social and psychological stressors such as stigma, isolation and concerns about persistence of symptoms (see [21,22]). Regardless of the cause of psychological symptoms, the literature agrees that these emotional disorders should be assessed and addressed.

Contrary to what can be expected, and despite the huge efforts that have been carried out to address emotional disorders in patients with other health conditions, long COVID-19 patients are not receiving appropriate psychological interventions. A recent systematic review has postulated that psychological interventions are more frequently provided to COVID-19 patients compared with long COVID-19 patients [23]. In this review, it was found that out of 19 psychological interventions available for COVID-19 populations, only 3 were focused on long COVID-19 patients (two of them were a case study) and none of them had a transdiagnostic approach [23].

Acknowledging the high prevalence rates of long COVID-19 conditions and the high presence of comorbid emotional disorders in this population, the main aim of this work is to explore the preliminary feasibility and clinical utility of the UP for the transdiagnostic treatment of emotional disorders in a sample of patients diagnosed with long COVID-19 condition. In this study we will report descriptive information from a sample of long COVID-19 patients (i.e., participants’ sociodemographic, medical and psychosocial profiles) as well results regarding participants’ satisfaction with the program. Additionally, we will also present changes in psychological variables scores following the application of the UP-based psychological intervention. Considering the findings observed in previous studies where the UP has been used to treat emotional disorders in samples with health conditions, we expect that individuals with long COVID-19 and emotional disorders participating in our pilot study will adhere to the intervention and rate the intervention program as satisfactory (primary outcomes). Finally, we also expect to obtain, after the UP intervention program, improvements in mental clinical diagnosis of emotional disorders, anxiety and depressive symptoms, difficulties in emotion regulation, dimensions of emotional disorders and quality of life (secondary outcomes), and those positive changes will be maintained at 6-month follow-up.

## 2. Materials and methods

### 2.1. Sample

Participants in this study were a subsample of a bigger project called ARACOV. The main aim of the ARACOV project was to characterize long COVID-19 patients and analyze the impact of a nutritional and rehabilitation intervention in their quality of life. Inclusion criteria for participation in the ARACOV project were having a documented COVID-19 infection and presenting long COVID-19 condition; being over 18 years old; presenting fatigue symptoms and having independent ambulation. Conversely, exclusion criteria include having severe neurological diseases; presenting respiratory insufficiency, rheumatic pathology or acute musculoskeletal injury; lacking Internet access; not being fluent in Spanish; having allergies to any of the components of the supplement provided in this project (e.g., fish or shellfish) or having received immunosuppressants or corticosteroids during the past weeks. Long COVID-19 patients that could not be included in the ARACOV study due to exclusion criteria were referred to the UP psychological program (for a detailed description of these procedures, see the protocol study [24]). Inclusion criteria to participate in this UP psychological transdiagnostic intervention were being an adult diagnosed with long COVID-19 condition (the diagnosis was established by the internal medicine physicians at the collaborating hospitals, based on a confirmed COVID-19 diagnosis and the duration of symptoms related to this condition), presenting emotional disorders or symptoms (Overall Anxiety Severity and Impairment Scale**,** OASIS scores ≥8 points and/or Overall Depression Severity and Impairment Scale, ODSIS scores ≥7 points), residing in the autonomous community of Aragón, be fluent in Spanish, having Internet access and signing the written informed consent. Exclusion criteria were participating in the nutritional and rehabilitation program, emotional disorders present before the COVID-19 infection, currently undergoing psychological/pharmacological treatment for mental disorders or presenting a severe mental health disorders (i.e., schizophrenia) or suicidal ideation.

**Fig 1:**
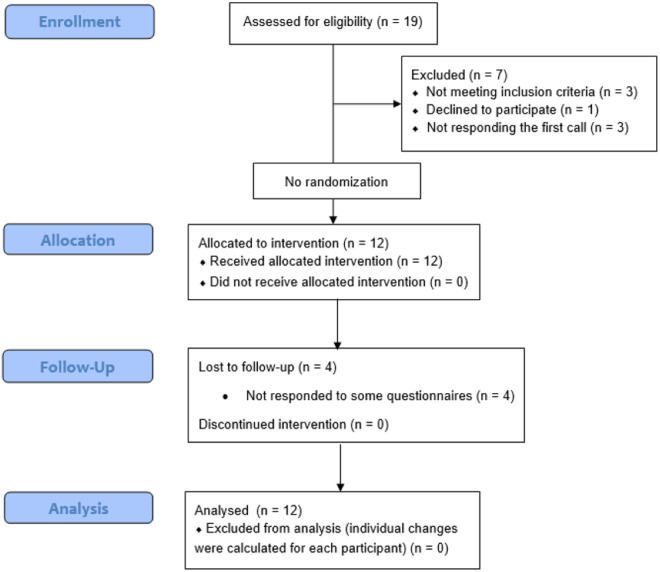
CONSORT 2010 Flow Diagram adapted to this single arm pilot study.

As can be found in Fig 1, in a first attempt 19 participants were assessed for eligibility. Of those, 7 participants were excluded for the following reasons: 3 did not meet the inclusion criteria, 1 declined to participate, and 3 did not respond to the initial contact. The remaining 12 participants met the inclusion criteria and agreed to participate and were finally allocated to the UP psychological intervention. All participants completed the 8 online sessions of the UP program but 4 of them did not respond to some of the questionnaires at follow-up. The participants of this single case pilot study were twelve long COVID-19 patients (mean age = 47.92; *SD* = 13.18) recruited from January 23, 2023 to December 29, 2023.

### 2.2. Procedures

This study and all its procedures were approved by the Research Ethics Committee of the Autonomous Community of Aragón (CEICA). For transparency purposes, this study was also registered in clinicaltrials.gov (NCT05581277) and a protocol study was published (see [24]). A multiple baseline design was proposed in the study protocol with a sample size of 60 participants (20 long COVID-19 patients in each baseline assessment condition). As defined by the American Psychological Association [25], a multiple baseline design is an experimental design that involves assessing one or more characteristics prior to the administration of an intervention in order to determine the initial state of the trait and to evaluate whether changes occur after the manipulation, specifically, after the administration of the psychological intervention. While our initial intention was to implement a multiple baseline study, challenges in participant recruitment made it unfeasible to randomly assigning participants to the three baseline conditions. Recruitment difficulties arose because, as previously mentioned, this study was part of a larger research project (ARACOV). Participants were initially assessed for eligibility within the ARACOV project, and only those who did not meet the inclusion criteria for that main study were then considered for inclusion in our study applying the UP psychological intervention. As a result, most individuals met the eligibility criteria for participation in the ARACOV project, leaving only a small number of participants who could be gradually enrolled in the UP study. Within this context, and for ethical reasons, we could not delay the start of the UP intervention until we had recruited the originally intended 60 participants, which would have allowed us to assign them across the three proposed experimental conditions (i.e., 6, 8, or 10 days of daily assessments prior to the start of the psychological intervention). Therefore, we decided to adapt the study design and conduct a pilot feasibility and clinical utility study. This approach allowed us to explore the preliminary usefulness and practical implementation of the psychological program before proposing a larger and more rigorous trial. For this reason, in the present study we are reporting results from a pilot study.

Participants meeting the inclusion criteria to participate in the UP program were given the information sheet and signed the written informed consent face-to-face with nurses working in the ARACOV project at three hospitals (Lozano Blesa Clinical University Hospital, Zaragoza; Huesca Pirineos Health Centre, and Teruel Ensanche Health Centre). Then, the psychologist responsible for conducting the UP program arranged an online session through video call to confirm the inclusion and exclusion criteria. The psychologist who conducted the initial assessments and delivered de UP psychological program was a post-doc psychologist with 8 years of experience in clinical psychology research, trained in the UP, and supervised by a UP certified psychologist. The supervisor is a psychologist with more than 20 years of experience in the assessment, diagnosis and treatment of emotional disorders in adults within a research context. During the initial session the Anxiety and Related Disorders Interview Schedule for DSM-5 (ADIS-5) [27] was administered to determine whether participants met the inclusion criteria. In cases where suicidal risk or severe mental disorders (i.e., personality disorders, schizophrenia) were suspected, participants underwent more in-depth assessment following DSM-5 criteria. The results of these evaluations were discussed with a clinical psychologist who is a member of the research group and has over 10 years of experience working in a public hospital. Once the inclusion criteria were confirmed, participants were sent by e-mail the pre-program assessment to be completed through the online platform *Google Forms*. After filling in the pre-program assessment, participants received the UP program individually throughout video calls. The online format was selected for conducting both the assessments and the psychological intervention because it facilitates the enrolment of all long COVID patients by reducing logistical and transportation barriers that might arise if the program were delivered in person.

### 2.3. Intervention

The UP program was composed of eight 1-hour individual online sessions delivered on a weekly basis. Each session addressed one of the original modules proposed in the second edition of the UP [10], maintaining all eight original modules, with each module being covered in a single session: 1 – setting goals and maintaining motivation; 2 – understanding emotions; 3 – mindful emotion awareness; 4 – cognitive flexibility; 5 – countering emotional behaviors; 6 – interoceptive exposures; 7 – emotion exposures; 8 – recognizing accomplishments and looking to the future. Core emotion regulation skills were trained from the third to the seventh session.

### 2.4. Measures

Assessment protocol included the evaluation of:

Sociodemographic and medical information:

- Sociodemographic and medical information: in the pre-assessment, participants answered sociodemographic questions that served to characterize the sample; they included sex, age, place of residence, marital status, employment, health habits and SARS CoV 2 vaccination.
- COVID-19 symptoms: to characterize the sample, in the pre-assessment, participants were asked about the COVID-19 symptoms they were experiencing in the present. Twenty-eight symptoms were arranged according to nine categories: general symptoms (e.g., weakness), respiratory symptoms (e.g., respiratory distress), gastrointestinal (e.g., nausea), musculoskeletal (e.g., joint pain), cutaneous (e.g., alopecia), otolaryngological (e.g., aphonia), neurological (e.g., headache), cardiovascular (e.g., thoracic pain), and others (free answer).

Primary outcomes-Feasibility measures:

- Client Satisfaction Questionnaire-8 (CSQ-8) [26]: An adaptation of this questionnaire was used to assess participants’ satisfaction with the intervention. The version employed in this study was composed of seven items (six original items from the CSQ-8 and an additional item to assess if participants felt some discomfort due to the intervention). Five open questions were also added to explore the qualitative opinion of participants with regards to inclusion or suppression of content, program’s duration, program’s format and a free question to allow participants to express any other concern related with the program.
- Satisfaction with the UP modules: the research team developed six questions to assess the perceived utility of each exercise practiced during the UP program (understanding emotions, mindful emotion awareness, cognitive flexibility, countering emotional behavior, interoceptive exposures, emotion exposures). An additional question was also included to explore general perceived utility of the UP. Higher scores indicate higher perceived utility of the UP for regulating emotions.

Secondary outcomes-Clinical utility:

- Anxiety and Related Disorders Interview Schedule for DSM-5 (ADIS-5) [27]: This structured clinical interview allows the assessment of different psychological disorders (i.e., anxiety, mood and related disorders) following the Diagnostic and Statistical Manual of Mental Disorders criteria (DSM-5) [28].
- Adjustment disorders: DSM-5 criteria [28] were used to assess the presence of adjustment disorders.
- Overall Anxiety Severity and Impairment Scale (OASIS) [29,30]: It evaluates the frequency, severity and interference of anxiety symptoms as well as avoidance behaviors. It is composed of five items (0 = no anxiety/never – 4 = extreme anxiety/always); total scores range from 0 to 20 points; higher scores indicate greater anxiety symptoms.
- Overall Depression Severity and Impairment Scale (ODSIS) [30,31]: It assesses the frequency, severity and interference of depressive symptoms as well as anhedonia. It consists of five items (0 = no depression/never – 4 = extreme depression/always) with total scores ranging from 0 to 20 points. Greater scores represent greater depressive symptoms.
- Difficulties in Emotion Regulation Scale (DERS) [32,33]: It measures emotion dysregulation when respondents have to cope with stressors. It is composed of 28 items (1 = almost never – 5 = almost always, reverse items are codified inversely); higher scores are indicative of greater emotion dysregulation. It is possible to obtain five dimensions of emotional dysregulation (inattention, confusion, rejection, interference, lack of control) as well as a global emotion dysregulation score, which is used in this study.
- Multidimensional Emotional Disorder Inventory (MEDI) [34,35]: It is a self-reported questionnaire that assess nine dimensions characteristic of emotional disorders: neurotic temperament, positive temperament, depressed mood, autonomic arousal, somatic anxiety, social anxiety, intrusive cognitions, traumatic re-experiencing and avoidance. It is composed of 48 items (0 = not characteristic of me – 8 = totally characteristic of me); higher scores represent higher presence of each dimension.
- Health-related quality of life (EuroQol) [36,37]: This questionnaire is composed of two separate components; the first component consists of five items that assesses five specific dimensions of self-perceived quality of life (EQ-5D). The second component is a Visual Analogue Scale (EQ-VAS) which assesses the subjective general health state of the patient using an item scored from 0 = worse imaginable health status to 100 = best imaginable heath status. Higher scores in this general perceived health item indicate greater quality of life. Given that recent studies have found that both scales could be useful when used separately and considering that the EQ-VAS component allows the global assessment of patients quality of life in a brief manner in healthcare settings [38], in our study we used only the EQ-VAS component.

***Time of assessments***: Sociodemographic and COVID-19 symptoms were assessed only in the pre-assessment. Satisfaction was only assessed in the post-assessment. The remaining measures were assessed at all assessment points (pre-assessment, post-assessment and 6-month follow-up). In the protocol study it was proposed to conduct four follow up sessions (at one, three, six and twelve months after the intervention). In this study, results derived from the 6 months’ follow-up assessment are presented. All assessments were conducted using the online platform *Google Forms,* and consequently, the research team was blinded to the participants’ responses.

### 2.5. Data analysis

Analyses were carried out with the SPSS software (version 25.0) [39]. Descriptive data (mean, standard deviation and proportions) of all study variables was calculated to characterize the sample. For variables with skewed distributions, the median and interquartile range (IQR) were also calculated to better characterize the sample. Second, feasibility results were analyzed (adherence rates and program satisfaction). We calculated adherence as a measure of acceptance of the intervention by calculating the proportion of attendance at sessions compared with programed sessions. Mann-Whitney U tests and chi-square tests were conducted to assess differences between participants who completed all assessment time points and those who did not. Additionally, satisfaction with the intervention was calculated with the answers to the CSQ-8 questionnaire (mean for each question) and open questions (additional modules needed, unnecessary content, program’s duration, satisfaction with online and individual formats and concerns about the program). Satisfaction mean with the UP modules are also reported.

Finally, due to the small sample size, the authors analyzed differences between assessment points individually. Thus, Reliable Change Index (RCI) [40] analyses served to explore whether changes in individual scores from pre-assessment to post-assessment and from pre-assessment to 6-month follow-up were statistically significant. Scores above ±1.96 points were considered to be significant. For the calculation of the RCI, we considered the normative data of the assessment instruments, which were extracted from the Spanish validations of each instrument (OASIS and ODSIS [30]; MEDI [35]; DERS [33]). The mean and standard deviation used to calculate the RCI for each instrument were as follows: OASIS = 3.02 (4.13); ODSIS = 2.79 (4.06); DERS = 58.4 (17.6). For the MEDI instrument, scores from its subscales were considered: Neurotic Temperament (NT) = 17.97 (8.6); Positive Temperament (PT) = 28.49 (6.51); Depressed Mood (DM) = 9.02 (8.46); Autonomic Arousal (AA) = 7.45 (7.74); Intrusive Cognitions (IC) = 9.98 (9.99); Somatic Anxiety (SOM) = 13.34 (7.68); Social Anxiety (SOC) = 13.88 (10.22); Traumatic Re-experiencing (TRM) = 7.11 (7.99); Avoidance (AVD) = 19.64 (10.93).

It was not possible to determine RCI for quality of life, as psychometric properties, mean and standard deviation of the EuroQol were not reported in a Spanish non-clinical sample. For this outcome, only tendencies to improve or worsen were reported comparing scores at different assessment points (Pre-Post and Pre-Follow up).

## 3. Results

### 3.1. Sociodemographic and medical characteristics of the sample

As can be found in Table 1, most participants were women (n = 11) and were in a stable romantic relationship (n = 10). A high proportion of participants (n = 9) had children (mean = 1.50; *SD* = 1.00; median = 2.00; IQR = 2.00). Half of the sample (n = 6) were currently employed while the remaining participants were on temporary sick leave (n = 5) or retired (n = 1). With regards to health habits, only 2 participants were smokers, none of them consumed alcoholic drinks and half of the participants (n = 7) exercised regularly (i.e., walk or pilates). In terms of COVID-19 characteristics, all participants had at least one vaccine against SARS CoV when the interview was conducted (mean = 2.08; *SD* = 0.90; median = 2.00; IQR = 0.50) and have been diagnosed with long COVID-19 for approximately one year (mean = 10.42 months; *SD* = 5.37). All participants reported having physical long COVID-19 symptoms (mean = 7.92; *SD* = 3.68), with the most frequent being asthenia (n = 11), arthralgia and myalgia (n = 10), memory and concentration issues (n = 9) and paresthesia (n = 8)

**Table 1:** Sociodemographic and medical profile of the participants.

Variable	Mean (*SD*); range n (proportion)
Age	47.92 (13.18); 25-77
Sex (women)	11 (91.7%)
Stable relationship	10 (83.3%)
No. children	1.5 (1.00) 0–3; median = 2 (IQR = 2.00)
Working	6 (50%)
**Health habits**	
Smoking (Yes)	2 (16.70%)
Alcohol (Yes)	0 (0%)
Exercise (Yes)	7 (58.30%)
No. vaccinations	2.08 (0.90); 1–4; median = 2.00 (IQR = 0.50)
Months since long COVID-19 diagnosis	10.42 (5.37); 6-21
**No. COVID-19 symptoms**	7.92 (3.68); 3-13
Asthenia	11 (91.67)
Arthralgia	10 (83.33)
Myalgia	10 (83.33)
Memory and concentration issues	9 (75)
Paresthesia (hand and/or feet)	8 (66.67)
Headache	7 (58.33)
Dyspnea (shortness of breath)	6 (50)
Vertigo	5 (41.67)
Chest pain	4 (33.33)
Loss of voice	3 (25)
Cough	3 (25)
Palpitations	2 (16.67)
Heartburn	2 (16.67)
Sweating	2 (16.67)
Dry skin	2 (16.67)
Abdominal pain	2 (16.67)
Alopecia	1 (8.33)
Odynophagia	1 (8.33)
Spatial disorientation	1 (8.33)
Weight loss	1 (8.33)
Nasal congestion	1 (8.33)
Palpitations	1 (8.33)
Menstrual irregularities	1 (8.33)
Diarrhea	1 (8.33)

### 3.2. Adherence to the intervention and satisfaction

Regarding adherence to the program, all participants attended the eight proposed sessions of the UP and the follow-up appointment at 6-month follow-up. However, adherence to the assessment protocol was lower. While all participants answered the online assessment at post-intervention, only eight participants answered the full follow-up assessment at 6 months, which means that 33% of participants dropped out of the assessment protocol. The results showed no statistically significant differences (*p* > .050) between participants who completed the 6-month assessment and those who did not in any clinical or sociodemographic variables, except for marital status (χ² = 4.80, df = 1, *p* = .028), with dropouts primarily being single participants (n = 2).

Results related to satisfaction with the psychological program are reported on Table 2. The program was considered to be of high quality and great utility, especially for solving problems adequately. All participants would recommend this program to a relative or friend and would participate in a similar program if needed in the future. High general satisfaction with the program was found (mean = 9.75) as well as with the UP modules (mean satisfaction ranged from 7.17 to 9 points).

**Table 2:** Participants’ satisfaction with the format and the content of the UP psychological program.

CSQ-8 Items (response range between 0 = low and 10 = high)	Mean
Program’s quality	9.75
Program’s utility	9.42
Recommendation of program	9.83
Program’s utility for solving problems	9.67
General satisfaction with the program	9.75
Intention to participate in a similar program if needed	9.83
Discomfort generated by the program (added)	1.42
Satisfaction with the modules (response range between 0 = low and 10 = high)	Mean
Program’s utility for regulating emotions in an adaptive way	8.42
Contents and skills’ utility for regulating emotions in an adaptive way: ARC	8.25
Mindful awareness	9.00
Cognitive flexibility	8.75
Identifying emotional behaviors	8.67
Describing opposed behaviors	8.67
Interoceptive exposures	7.17
Emotional exposures	7.75

Some qualitative reports were also obtained from open questions. In relation to the inclusion of other *additional content* some participants recommended sharing experiences with other COVID-19 patients and including more information about pain units, assertiveness, and management of emotions in different personal areas (i.e., work). Six patients considered the psychological program was complete and did not propose any additional content. Participants were also asked about *unnecessary content*. All participants agreed that all modules taught with the UP were useful and they did not suggest eliminating any of them. Only one participant stated that this program should be more personalized to each participant, as not everyone may need all the modules. Regarding *program duration* (eight 1-hour sessions), seven participants considered it was adequate but four also proposed that it could have more sessions. In terms of frequency of sessions, only one participant expressed the desire to have biweekly sessions. Additionally, all participants were satisfied with the *online (i.e., videocalls) and individual format*. With respect to online advantages, some participants said they were more comfortable, they could be adapted to the participant’s needs, avoided having to travel long distances to the hospital or reduced the physical difficulties caused by having to get out of their home. Finally, no *concerns about the program* were mentioned. All participants reported high adherence with the psychologist and expressed feeling listened to and understood.

### 3.3. Change in mental clinical diagnosis

As shown in Table 3, before the psychological intervention, participants met the criteria for major depression (n = 8), agoraphobia (n = 6), general anxiety (n = 4), panic disorder (n = 3), adjustment disorder (n = 2), social anxiety (n = 1) and non-specified anxiety disorder (n = 1). Comorbidity was present in 8 participants. Of those, 3 participants were diagnosed with 2 comorbid psychological disorders and 5 participants had 3 comorbid disorders.

**Table 3:** Mental clinical diagnosis across assessment points.

ID	Mental clinical diagnosis at pre-assessment	Mental clinical diagnosis at post-assessment	Mental clinical diagnosis at follow-up
1	Adjustment disorder mixed symptoms (anxiety and depressive symptoms).	No longer meeting criteria.	No longer meeting criteria.
2	General anxiety disorder.	No longer meeting criteria.	No longer meeting criteria.
3	Major depression, panic disorder, agoraphobia.	Major depression.	Major depression.
4	Major depression, agoraphobia, social anxiety.	Social anxiety.	No longer meeting criteria.
5	Adjustment disorder mixed symptoms (anxiety and depressive symptoms).	No longer meeting criteria.	No longer meeting criteria.
6	Major depression, agoraphobia, general anxiety disorder.	Major depression.	Major depression.
7	Major depression, generalized anxiety disorder.	Generalized anxiety disorder.	Generalized anxiety disorder.
8	Major depression, panic, agoraphobia.	Major depression.	No longer meeting criteria.
9	Major depression, agoraphobia.	No longer meeting criteria.	No longer meeting criteria.
10	Major depression, panic disorder.	No longer meeting criteria.	No longer meeting criteria.
11	Major depression, agoraphobia, non-specified anxiety disorder.	Major depression.	Major depression.
12	General anxiety disorder.	No longer meeting criteria.	No longer meeting criteria.

Six participants no longer met criteria for mental clinical diagnosis after the intervention. On the other hand, four participants continued to experience major depression, one still met criteria for social anxiety and one still presented generalized anxiety disorder after the intervention. Of those participants, one had recovered from major depression by the 6-month follow-up, and another had recovered from social anxiety. Thus, at the 6-month follow-up, only 3 participants met criteria for major depression (at the beginning of the program they had 3 comorbid psychological disorders) and one met criteria for generalized anxiety disorder (before the intervention, this participant was diagnosed with 2 comorbid disorders).

### 3.4. Evolution in therapeutic objectives across assessment points

The first session of the program consisted of establishing therapeutic objectives to be achieved once participants had learned all the emotion regulation skills of the program. A summary of the proposed objectives for each participant is reported on Table 4. A total of 36 objectives were proposed in the first session (three objectives for each participant). Of those, 39% (n = 14) of the therapeutic objectives had been achieved when the program ended (post-assessment), 39% (n = 14) of the objectives were initiated but not completed (in progress) and only 22% (n = 8) of the objectives had not been started at post-assessment. At 6-month follow-up, 72% (n = 26) of the therapeutic objectives had been achieved, 14% (n = 5) were in progress, 8% (n = 3) had not been started and 6% (n = 2) had been changed (participants had changed the type of exercise they would like to practice).

**Table 4:** Achievement of therapeutic objectives across assessment points.

ID	Objectives proposed at the beginning of the intervention	Evolution at Post-assessment	Evolution at Follow-up
1	To start labor courses (currently on an absolute incapacity for work).	In progress.	Achieved.
	To talk about their past job.	Achieved.	Achieved.
	To go to their past job.	In progress.	Achieved.
2	To use more assertive communication skills with relatives.	In progress.	Achieved.
	To start to go out for a walk daily.	Achieved.	Achieved.
	To resume excursions to a nearby town once a month.	In progress.	Achieved.
3	To go out with daughters alone twice a week.	Achieved.	Achieved.
	To go to the gym twice a week.	Achieved.	Achieved.
	To go out with friends at least once every two weeks.	In progress.	In progress.
4	To do pilates once a week.	Not started.	Changed.
	To have their children’s friends stay over at home.	In progress.	Achieved.
	To greet friends she meets on the street.	In progress.	Achieved.
5	To go to their country twice a month.	Achieved.	Achieved.
	To start to learn English.	Achieved.	Achieved.
	To go out for a bike ride once a week.	Not started.	Changed.
6	To talk about their emotional suffering and stopping working due to long COVID-19 symptoms.	Not started.	Not started.
	To resume making crafts with friends once a week.	Achieved.	Achieved.
	To go out with their nephews.	Achieved.	Achieved.
7	To use more assertive communication skills with her daughters.	Achieved.	Achieved.
	To share household chores with daughters and husband.	In progress.	In progress.
	To return to work.	Not started.	Achieved.
8	To join a pleasure activity once a week (i.e., Zumba)	Not started.	Not started.
	To meet with friends once a week.	In progress.	Achieved.
	To express their opinion while with co-workers during the snack break.	In progress.	In progress.
9	To spend more time playing games with their children.	Achieved.	Achieved.
	To use more assertive communication skills in their job.	In progress.	Achieved.
	To take the bus to work again.	In progress.	Achieved.
10	To go out with friends once a week.	In progress.	Achieved.
	To reduce self-demand: To practice cognitive flexibility when finding difficulties at work.	Achieved.	Achieved.
	To establish a routine to do housework daily.	Achieved.	Achieved.
11	To go to the festivities of her town with her daughters.	Achieved.	Achieved.
	To resume driving a car twice a week.	Not started.	In progress.
	To resume work activities (i.e., preparing materials for children with language difficulties).	Not started.	Not started.
12	To reduce the number of times she checks her notes at work.	In progress.	Achieved.
	To start running once a week and swimming once a week again.	Not started.	In progress.
	To use more assertive communication skills with her children.	Achieved.	Achieved.

*Note*: ID = Participant Identification.

### 3.5. Reliable change index by participants

#### 3.5.1. Changes in anxiety symptoms (OASIS scores).

As can be observed in Table 5, all participants in our study, except for participant number 5, had higher anxiety scores (OASIS) than normative population at pre-assessment. Additionally, eight participants had clinical/subclinical symptoms of anxiety as established by our cut-off (scores in OASIS≥8 points). As will be shown in the next section, participants who did not present clinical symptoms of anxiety at pre-assessment (participants number 2, 5, 9 and 10) were included in the study because they had clinical symptoms of depression.

**Table 5:** Changes in anxiety, depression and emotion dysregulation across assessment points.

OASIS	ID	Pre	Post	6M-FU	ND (SD)	RCI Pre-Post	Change	RCI Pre-6M	Change
	1	13	0	2	3.92 (4.13)	**−9,09**	Improvement	**−7,69**	Improvement
	2	7	4	13		**−2,10**	Improvement	**4,20**	Deterioration
	3	10	10	4		0,00		**−4,20**	Improvement
	4	8	5	2		**−2,10**	Improvement	**−4,20**	Improvement
	5	1	0	8		−0,70		**4,90**	Deterioration
	6	9	15	0		**4,20**	Deterioration	**−6,29**	Improvement
	7	14	10	0		**−2,80**	Improvement	**−9,79**	Improvement
	8	12	2	2		**−6,99**	Improvement	**−6,99**	Improvement
	9	6	1	3		**−3,50**	Improvement	**−2,10**	Improvement
	10	6	0	–		**−4.69**	Improvement	–	
	11	10	10	8		0,00		−1.40	
	12	10	3	2		**−4,90**	Improvement	**−5.59**	Improvement
ODSIS	ID	Pre	Post	6M-FU	ND (SD)	RCI Pre-Post	Change	RCI Pre-6M	Change
	1	10	0	1	2.79 (4.06)	**−7,81**	Improvement	**−7,03**	Improvement
	2	8	5	14		**−2,34**	Improvement	**4,69**	Deterioration
	3	9	11	6		1,56		**−2,34**	Improvement
	4	9	4	0		**−3,91**	Improvement	**−7,03**	Improvement
	5	8	0	7		**−6,25**	Improvement	−0,78	
	6	6	12	0		**4,69**	Deterioration	**−4,69**	Improvement
	7	14	10	0		**−3,13**	Improvement	**−10,94**	Improvement
	8	10	2	2		**−6,25**	Improvement	**−6,25**	Improvement
	9	9	0	2		**−7,03**	Improvement	**−5,47**	Improvement
	10	7	0	–		**−5.47**	Improvement	–	
	11	10	9	7		−0,78		**−2.34**	Improvement
	12	10	0	0		**−7,81**	Improvement	**−7.81**	Improvement
DERS	ID	Pre	Post	6MFU	ND (SD)	RCI Pre-Post	Change	RCI Pre-6M	Change
	1	48	41	36	58,4 (17,6)	−1,06		−1,82	
	2	65	79	63		**2,13**	Deterioration	−0,3	
	3	85	53	–		**−4,86**	Improvement	–	
	4	95	76	53		**−2,89**	Improvement	**−6,38**	Improvement
	5	55	45	57		−1,52		0,3	
	6	101	110	–		1,37		–	
	7	97	80	–		**−2,58**	Improvement	–	
	8	83	92	88		1,37		0,76	
	9	43	33	41		−1,52		−0,3	
	10	52	54	–		0,3		–	
	11	80	62	57		**−2,74**	Improvement	**−3,5**	Improvement
	12	37	42	42		0,76		0,76	

*Note*: OASIS = Overall Anxiety Severity and Impairment Scale; ODSIS = Overall Depression Severity Impairment Scale; DERS = Difficulties in Emotion Regulation Scale; ID = Participant identification; Pre = pre-assessment; Post = Post-assessment; 6M-FU = 6 months’ follow-up; ND = Normative Data; SD = Standard Deviation; RCI = Reliable Change Index [Statistically significant changes are indicated in bold (>1.96)]. OASIS normative data obtained from reference [30]; ODSIS normative data obtained from reference [30]; DERS normative data obtained from reference [33].

OASIS scores significantly decreased from pre-assessment to post-assessment in eight participants (participants number 1, 2, 4, 7, 8, 9, 10, 12; RCI ranged from −2.10 to −9.09). One participant reported significant increased anxiety scores at post-assessment (participant number 6; OASIS_pre_ = 9 - OASIS_post_ = 15; RCI = 4.20).

Differences between outcomes at pre-assessment and 6-month follow-up revealed that six participants maintained the improvement in anxiety scores in the mid-term (participants number 1, 4, 7, 8, 9, 12; RCI ranged from −2.10 to −9.79). Additionally, two participants that had not shown improvements in the post-assessment, reported decreased anxiety scores in the 6-month follow-up assessment (participants 3 and 6; RCI = −4.20 and −6.29, respectively). In contrast, anxiety scores significantly worsened in the follow-up assessment in two participants (participants 2 and 5, RCI = 4.20 and 4.90, respectively).

#### 3.5.2. Changes in depressive symptoms (ODSIS scores).

As shown in Table 5, all participants in our study had higher depressive scores at pre-assessment than normative populations and all of them, except for participant number 6, had clinical/subclinical symptoms of depression as established by our cut-off score (ODSIS≥7).

When comparing scores from pre- to post-assessment, we found that depressive scores significantly decreased in nine participants (participants number 1, 2, 4, 5, 7, 8, 9,10, 12; RCI ranged from −2.34 to −7.81). Depressive scores significantly worsened in one participant at post-assessment (participant 6; ODSIS_pre_ = 6 - ODSIS_post_ = 12; RCI = 4.69).

Seven participants had maintained the improvements in their depressive scores at 6-month follow-up (participants number 1, 4, 7, 8, 9, 11, 12; RCI between −2.34 to −10.94). Two additional participants who had not found relief for depressive scores by the post-assessment had improved by the 6-month follow-up (participants 3 and 6; RCI −2.34 and −4.69 respectively). As with anxiety symptoms, participant 2’s depressive scores had worsened at 6-month follow-up; the aggravation of depressive outcomes in this participant were clinically significant (RCI = 4.69).

#### 3.5.3. Changes in emotion dysregulation (DERS scores).

Table 5 shows emotion dysregulation scores at pre-assessment and changes across assessment points. Before the intervention, seven participants (participants number 2, 3, 4, 6, 7, 8 and 11) had worse scores in emotion dysregulation than the normative population. After the program, four of these participants had significantly improved their scores in emotion dysregulation (participants 3, 4, 7, 11; RCI between −2.58 and −4.86) and one participant had significantly worsened in emotion dysregulation scores (participant number 2; DERS_pre_ = 65 – DERS_post_ = 79; RCI = 2.13).

Only four of these participants with high emotion dysregulation at pre-assessment responded to the follow-up assessment, and only two of them maintained significant improvements in emotion dysregulation scores (participants 4 and 11; RCI = −6.38 and −3.5, respectively).

As indicated in Table 5, the remaining five participants had emotion dysregulation scores similar or lower than the normative population at pre-assessment (participants number 1, 5, 9, 10, 12) and significant changes were not found neither at the post-assessment nor at the 6-month follow-up (RCI<±1.96).

#### 3.5.4. Changes in the emotional disorders’ dimensions profile (MEDI scores).

For readability purposes, only significant changes in the scores of emotional disorder dimensions measured with MEDI are reported in the next lines (for a detailed description of these results, see Table 6).

**Table 6:** Changes in the Multidimensional Emotional Disorder Inventory across the intervention.

Neurotic Temperament (NT)	ID	Pre	Post	6M-FU	ND (SD)	RCI Pre-Post	Change	RCI Pre-6M	Change
	1	13	6	6	17.97 (8.6)	−1,29		−1,29	
	2	25	29	29		0,74		0,74	
	3	17	11	–		−1,1		–	
	4	39	33	32		−1,1		−1,29	
	5	16	14	18		−0,37		0,37	
	6	25	21	–		−0,74		–	
	7	25	38	–		**2,39**	Deterioration	–	
	8	32	25	31		−1,29		−0,18	
	9	31	7	15		**−4,41**	Improvement	**−2,94**	Improvement
	10	17	21	–		0,74		–	
	11	19	22	19		0,55		0	
	12	16	18	7		0,37		−1,65	
Positive Temperament (PT)	ID	Pre	Post	6M-FU	ND (SD)	RCI Pre-Post	Change	RCI Pre-6M	Change
	1	25	25	34	28.49 (6.51)	0		1,92	
	2	19	22	22		0,64		0,64	
	3	29	34	–		1,07		–	
	4	12	16	18		0,85		1,28	
	5	20	22	22		0,43		0,43	
	6	19	13	–		−1,28		–	
	7	22	31	–		1,92		–	
	8	27	35	39		1,71		**2,56**	Improvement
	9	27	33	31		1,28		0,85	
	10	26	31	–		1,07		–	
	11	26	20	30		−1,28		0,85	
	12	35	35	35		0		0	
Depressed Mood (DM)	ID	Pre	Post	6MFU	ND (SD)	RCI Pre-Post	Change	RCI Pre-6M	Change
	1	11	4	2	9.02 (8.46)	−1,62		**−2,09**	Improvement
	2	16	18	13		0,46		−0,7	
	3	27	22	–		−1,16		–	
	4	28	22	11		−1,39		**−3,94**	Improvement
	5	15	9	6		−1,39		**−2,09**	Improvement
	6	29	35	–		1,39		–	
	7	15	19	–		0,93		–	
	8	11	4	13		−1,62		0,46	
	9	12	0	13		**−2,78**	Improvement	0,23	
	10	29	15	–		**−3,25**	Improvement	–	
	11	18	16	10		−0,46		−1,86	
	12	9	7	3		−0,46		−1,39	
Automatic Arousal (AA)	ID	Pre	Post	6M-FU	ND (SD)	RCI Pre-Post	Change	RCI Pre-6M	Change
	1	18	2	2	7.45 (7.74)	**−3,19**	Improvement	**−3,19**	Improvement
	2	9	15	11		1,2		0,4	
	3	27	22	–		−1		–	
	4	11	16	7		1		−0,8	
	5	0	1	0		0,2		0	
	6	25	26	–		0,2		–	
	7	19	26	–		1,39		–	
	8	12	4	8		−1,59		−0,8	
	9	1	0	8		−0,2		1,39	
	10	6	5	–		−0,2		–	
	11	23	12	13		**−2,19**	Improvement	**−1,99**	Improvement
	12	2	9	3		1,39		0,2	
Intrusive Cognitions (IC)	ID	Pre	Post	6M-FU	ND (SD)	RCI Pre-Post	Change	RCI Pre-6M	Change
	1	2	0	1	9.98 (9.99)	−0,39		−0,2	
	2	14	17	16		0,59		0,39	
	3	7	18	–		**2,16**	Deterioration	–	
	4	20	23	14		0,59		−1,18	
	5	0	0	0		0		0	
	6	33	33	–		0		–	
	7	24	40	–		**3,14**	Deterioration	–	
	8	3	20	22		**3,34**	Deterioration	**3,73**	Deterioration
	9	1	0	0		−0,2		−0,2	
	10	1	0	–		−0,2		–	
	11	24	8	4		**−3,14**	Improvement	**−3,93**	Improvement
	12	0	0	0		0		0	
Somatic Anxiety (SOM)	ID	Pre	Post	6MFU	ND (SD)	RCI Pre-Post	Change	RCI Pre-6M	Change
	1	8	7	7	13.34 (7.68)	−0,18		−0,18	
	2	25	22	20		−0,55		−0,92	
	3	28	32	–		0,74		–	
	4	10	16	13		1,1		0,55	
	5	20	16	17		−0,74		−0,55	
	6	34	35	–		0,18		–	
	7	19	35	–		**2,95**	Deterioration	–	
	8	21	40	40		**3,5**	Deterioration	**3,5**	Deterioration
	9	6	4	8		−0,37		0,37	
	10	13	7	–		−1,1		–	
	11	24	21	19		−0,55		−0,92	
	12	2	5	4		0,55		0,37	
Social Anxiety (SOC)	ID	Pre	Post	6M-FU	ND (SD)	RCI Pre-Post	Change	RCI Pre-6M	Change
	1	9	7	2	13.88 (10.22)	−0,49		−1,72	
	2	28	26	27		−0,49		−0,25	
	3	21	10	–		**−2,7**	Improvement	–	
	4	40	33	18		−1,72		**−5,39**	Improvement
	5	4	2	4		−0,49		0	
	6	37	36	–		−0,25		–	
	7	21	18	–		−0,74		–	
	8	14	25	38		**2,7**	Deterioration	**5,88**	Deterioration
	9	5	0	5		−1,23		0	
	10	19	7	–		**−2,94**	Improvement	–	
	11	24	27	32		0,74		1,96	
	12	3	8	5		1,23		0,49	
Traumatic Re-experiencing (TRM)	ID	Pre	Post	6M-FU	ND (SD)	RCI Pre-Post	Change	RCI Pre-6M	Change
	1	1	0	0	7.11 (7.99)	−0,25		−0,25	
	2	2	7	6		1,23		0,98	
	3	1	1	–		0		–	
	4	18	21	15		0,74		−0,74	
	5	0	0	0		0		0	
	6	23	24	–		0,25		–	
	7	18	36	–		**4,42**	Deterioration	–	
	8	23	24	18		0,25		−1,23	
	9	13	3	7		**−2,46**	Improvement	−1,47	
	10	0	0	–		0		–	
	11	1	0	0		−0,25		−0,25	
	12	0	0	0		0		0	
Avoidance (AVD)	ID	Pre	Post	6MFU	ND (SD)	RCI Pre-Post	Change	RCI Pre-6M	Change
	1	24	9	15	19.64 (10.93)	−**2,02**	Improvement	−1,21	
	2	39	27	31		−1,62		−1,08	
	3	32	40	–		1,08		–	
	4	36	41	37		0,67		0,13	
	5	11	1	0		−1,35		−1,48	
	6	42	43	–		0,13		–	
	7	29	53	–		**3,24**	Deterioration	–	
	8	31	42	45		1,48		1,89	
	9	10	0	10		−1,35		0	
	10	16	14	–		−0,27		–	
	11	34	24	28		−1,35		−0,81	
	12	13	23	6		1,35		−0,94	

*Note*: MEDI = Multidimensional Emotional Disorder Inventory; ID = Participant identification; Pre = pre-assessment; Post = Post-assessment; 6M-FU = 6 months’ follow-up; ND = Normative Data; SD = Standard Deviation; RCI = Reliable Change Index [Statistically significant changes are indicated in bold (>1.96)]. MEDI normative data obtained from reference [35].

Seven participants scored higher for **Neurotic Temperament** (NT) at pre-assessment than normative populations (participants 2, 4, 6, 7, 8, 9, 11). At post-assessment, NT scores had significantly improved in participant number 9 (NT_pre_ = 31 – NT_post_ = 7; RCI = −4.41). This improvement was also maintained at the 6-month follow-up (RCI = −2.94). On the other hand, participant number 7 had significantly worse scores in NT at post-assessment (NT_pre_ = 25 – NT_post_ = 38; RCI = 2.39) and did not answer the follow-up assessment.

Ten participants had lower scores in **Positive Temperament** (PT) than normative populations (participants number 1, 2, 4, 5, 6, 7, 8, 9, 10, 11). At post-assessment, PT outcomes had not significantly improved in any of the participants. Only participant number 8 obtained an increase in PT scores at the 6-month follow-up (RCI = 2.56).

The scores for **Depressed Mood** (DM) at pre-assessment were higher than those obtained by normative populations for all participants, except for participant number 12. A significant reduction in DM scores was observed at post-assessment in two participants (participants number 9 and 10; RCI −2.78 and −3.25, respectively). However, participant number 9 had not maintained this improvement at the 6-month follow-up (RCI = 0.23) and participant number 10 did not answer the follow-up assessment. Three participants had not reduced DM outcomes at post-assessment but they actually had at the 6-month follow-up (participants 1, 4 and 5; RCI between −2.09 and −3.94).

**Autonomic Arousal** (AA) scores were high in eight participants at pre-assessment (participants number 1, 2, 3, 4, 6, 7, 8, 11 had higher AA scores than normative population). Two participants found significant relief from AA levels at post-assessment (participants 1 and 11; RCI −3.19 and −2.19, respectively) and both had maintained these improvements at the 6-month follow-up (RCI −3.19 and −1.99, respectively).

Five participants had higher scores in **Intrusive Cognitions** (IC) at pre-assessment than those obtained by normative populations (participants number 2, 4, 6, 7, 11). IC was lower at post-assessment for participant number 11 (IC_pre_ = 24 – IC_post_ = 8; RCI = −3.14) and this was maintained at the 6-month follow-up (IC_follow-up_=4; RCI = −3.93). However, three participants had a significant increase in their IC scores at post-assessment (participants number 3, 7 and 8; RCI between 2.16 and 3.14). Of those, only participant 8 answered the follow-up assessment and maintained a high IC score (IC_pre_ = 3 – IC_follow-up_=22; RCI = 3.73).

Seven participants scored higher in **Somatic Anxiety** (SOM) than normative populations (participants number 2, 3, 5, 6, 7, 8, 11). SOM scores did not significantly improve at post-assessment for any of these participants while they significantly worsened for participant 7 (SOM_pre_ = 19 – SOM_post_ = 35; RCI = 2.95) and participant 8 (SOM_pre_ = 21 – SOM_post_ = 40; RCI = 3.5). While participant 7 did not answer the 6-month follow-up assessment, participant 8 maintained this deterioration at 6 months follow-up (SOM_pre_ = 21 – SOM_post_ = 40; RCI = 3.5).

**Social Anxiety** (SOC) scores were high at pre-assessment in eight participants (participants number 2, 3, 4, 6, 7, 8, 10, 11). At post-assessment, SOC levels were lower in two participants (participants number 3 and 10, RCI −2.7 and −2.94, respectively) but they did not answer the follow-up assessment. One participant improved their SOC scores but only at the 6-month follow-up (participant number 4, SOC_pre_ = 40 – SOC_follow-up_=18, RCI = −5.39). Compared with pre-assessment, one participant (number 8) got significantly worse scores in SOC at the post-assessment (RCI = 2.7) and follow-up assessment (RCI = 5.88).

The scores for **Traumatic Re-experiencing** (TRM) at pre-assessment were higher than those obtained by normative populations in five participants (participants number, 4, 6, 7, 8, 9). TRM scores were reduced at post-assessment for one participant (number 9; TRM_pre_ = 13 – TRM_post_ = 3, RCI = −2.46), however, improvements were not significantly maintained at the 6-month follow up (TRM_follow-up_=7, RCI = −1.47). One participant got significant higher scores in TRM at post-assessment compared with their scores at pre-assessment (participant 7, TRM_pre_ = 18 - TRM_post_ = 36, RCI = 4.42). This participant did not answer the follow-up assessment.

**Avoidance** (AVD) was high at pre-assessment in eight participants (participants number 1, 2, 3, 4, 6, 7, 8, 11). AVD scores at post-assessment had significantly improved in participant 1 (AVD_pre_ = 24 – AVD_post_ = 9, RCI = −2.02) and had significantly worsened in participant 7 (AVD_pre_ = 29 – AVD_post_ = 53, RCI = 3.24). However, participant number 1 had not maintain the improvement at the 6-month follow-up (RCI = −1.21) and participant 7 did not answer the follow-up assessment.

#### 3.5.5. Changes in quality of life.

**Fig 2:**
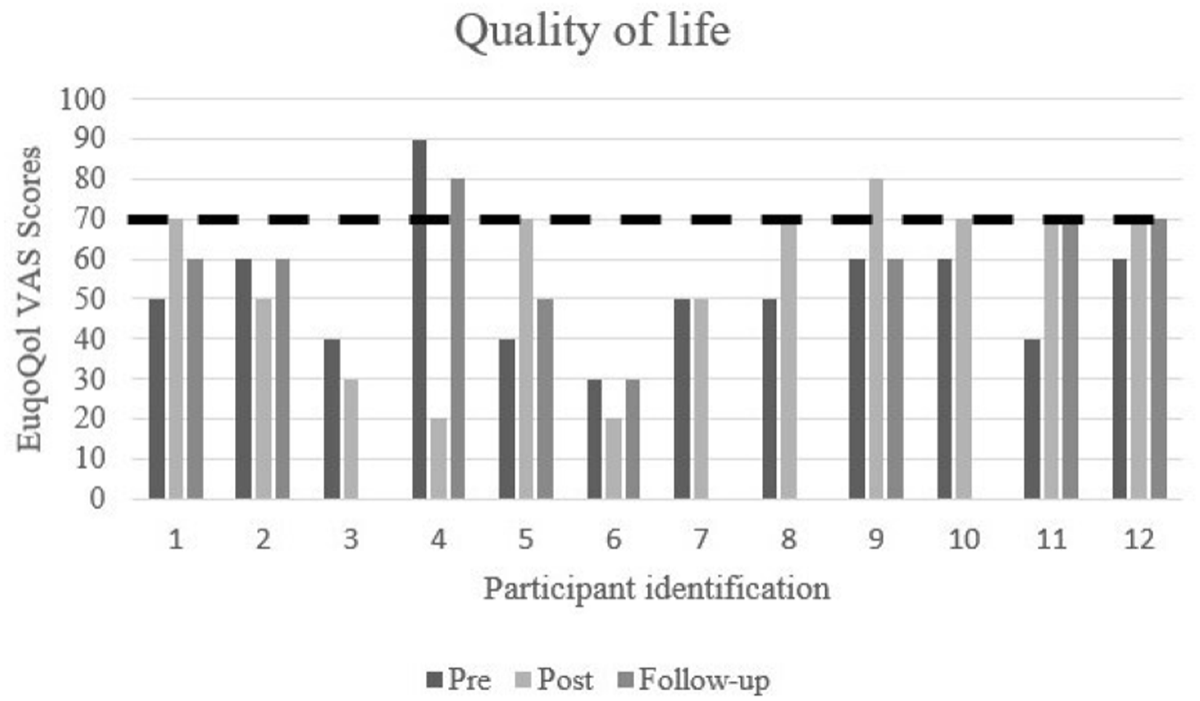
Changes in quality of life from pre-assessment to 6-month follow-up. The dashed line indicates scores obtained by normative population.

As shown in Fig 2, except for participant number 4, at pre-assessment all participants had low quality of life scores (between 30 and 60 points) compared with the normative population (scores of around 70 points). An improvement in quality of life levels at post-assessment was observed in seven participants (participants number 1, 5, 8, 9, 10, 11, 12) who in this assessment point obtained scores in quality of life similar to normative populations (represented by a dashed line in Fig 2). Two of these participants had maintained their quality of life levels at the 6-month follow-up (participants 11 and 12) while one participant had returned to their pre-assessment scores by the 6-month follow-up (participant 9). Four participants had not improved their quality of life scores at neither the post-assessment nor the 6-month follow-up (participants 2, 3, 4, 6) and one participant maintained moderated quality of life scores across assessment points (participant number 7).

## 4. Discussion

The main aim of this pilot study was to explore the preliminary feasibility and clinical utility of the UP for the transdiagnostic treatment of emotional disorders in long COVID-19 patients. Our hypothesis about the feasibility of the program was confirmed, as high levels of adherence and satisfaction with the program were observed.

In terms of UP feasibility in long COVID-19 patients, all participants attended the eight proposed sessions of the UP and the additional follow-up session, although adherence to the assessment protocol was quite low. All participants answered the questionnaires at pre- and post-assessment points. However, four participants failed to answer the questionnaire at the 6-month follow-up. Previous studies have revealed high attrition rates when longitudinal online assessments are conducted [41]. Possible solutions to reduce dropout in assessment protocol could include reducing the number and length of questionnaires, allowing participants to answer the questionnaires at the end of the last session or facilitating oral responses (i.e., through automated voicemail).

Another important feasibility outcome explored in this work was satisfaction with the intervention. Results from our pilot study, and similar to previous research using the UP in general populations [42] and in medical conditions [14], indicated that the UP is a well-accepted, high quality and useful psychological intervention. According to the feedback provided by our participants, some of the improvements to be considered in future studies which apply the UP to long COVID-19 patients are favoring peer contact to share information and experiences and increasing the number of sessions. Group sessions have been used in previous health conditions [14,43] and also with COVID-19 patients [44] so we propose their use with long COVID-19 patients to facilitate peer support and to help extend the number of sessions with minimal impact in the program cost-effectiveness. Moreover, given that participants in our study were satisfied with the online format, these group sessions could be conducted online, for instance through videocalls, to reach participants that cannot attend to face-to-face sessions (i.e., patients living in rural areas or with severe physical symptoms that prevent them from travelling long distances to the hospital) [45].

Some of the suggestions provided by participants in the UP psychological intervention to improve the program included adding information about pain management units and incorporating additional contents not included in the UP, such as training in assertive communication skills. In previous studies, long COVID-19 patients have reported difficulties in accessing information about long COVID-19 units and have emphasized the need to create specialized services that facilitate collaborative work among professionals [46]. These findings highlight the need to continue improving healthcare services, especially when dealing with novel illnesses that involve complex and uncertain management. With regards to training in assertive communication skills, patients with long COVID often face situations of stigma and discrimination [47,48]. In a recent qualitative study, patients with long COVID-19 reported feelings of stigmatization due to negative comments or attitudes from others toward their experience and recovery process [49]. In this context, people with long COVID-19 may experience difficulties in responding to such comments, highlighting the potential value of communication skills training. Previous studies have incorporated assertive communication training when applying the UP in medical conditions [50]. Therefore, future research should explore the utility of including such communication skills in interventions for patients with long COVID-19.

Regarding the hypothesis related to the clinical utility of the intervention, not all of them were confirmed. On the one hand, promising results have been obtained in our pilot study in terms of reduction in mental clinical diagnosis, achievement of therapeutic goals, reduction in anxiety and depressive scores. On the other hand, modest results have been found in the improvement of emotion dysregulation scores, emotional dimensions of the emotional disorders and quality of life outcomes. In the next lines we will briefly discuss some of the main results related to the clinical utility of our pilot study.

Results derived from our study support previous studies that found a worrisome physical and emotional burden in long COVID-19 patients [18,19,51]. The physical impact of long COVID-19 can be observed in the great number of physical symptoms that were present in our sample before the UP psychological program. Long COVID-19 physical symptoms have been associated with emotional suffering in those patients [52,53]. Thus, this study offers preliminary insights into how the psychological burden experienced by long COVID-19 patients (i.e., anxiety and depressive symptoms) might potentially be alleviated.

The emotional burden of chronic physical conditions has been reported in previous studies [6] and it is also observed in our study by the 25 clinical diagnoses that were established previous to the implementation of the UP with important comorbidity rates. In terms of improvements in psychological disorders, 75% of these diagnoses were no longer present at post-assessment, and 84% of them at 6-month follow-up (any participant presented comorbidity at the follow-up assessment). The observed reduction not only in the amount of clinical diagnoses but also in comorbidity rates suggest that transdiagnostic interventions such as the UP may be useful to address simultaneous psychological disorders [13,54]. While acknowledging the potential use of the UP in long COVID-19 patients, we should also recognize that four participants still met criteria for one mental clinical diagnosis at the 6-month follow-up. Although a reduction in comorbidity (from 3 diagnoses at pre-assessment to 1 diagnose at follow-up) was observed in these participants, results from our study may indicate that most severe cases (e.g., those who present more than two clinical diagnoses) could be more resistant to treatment and eight sessions of psychological treatment may be insufficient. Future studies with larger samples should analyze what the profile is of participants that may be resistant to change and may propose solutions for those particular cases (i.e., increasing the number of sessions, reinforcing some modules according to the severity of the symptoms, adding different modules).

Apart from focusing on a reduction in mental clinical diagnosis, tendencies to improve over the course of the intervention can also be evaluated by exploring whether participants achieve their personal goals. One of the main objectives of the UP is to train participants in emotion regulation skills to tolerate unpleasant emotions and achieve their personal goals or therapeutic objectives, despite feeling some discomfort in the process [10]. In this regard, most of the participants in our study achieved their personal goals at post-assessment and only 22% of the proposed objectives had not been started when the interventions ended. It is reasonable that not all the therapeutic objectives had been completed at the end of the intervention, because in our study the UP program only lasted eight sessions and unhelpful emotion regulation skills that have been used for years may need more time to be modified. For this reason, from the beginning of the intervention, and especially when the program has ended, it is important that therapists remind participants that more practice is needed to achieve their goals [55] and encourage participants to continue practicing adaptive emotion regulations skills in everyday situations.

Across all the variables that were assessed in our study, the most noticeable improvements were found in anxiety and depressive scores. A recent systematic review have also found some relief in anxiety and depressive symptoms when psychological interventions were provided in long COVID-19 participants [56]. In the specific case of the UP, results from our study, and similarly with previous studies applying the UP in participants suffering health conditions (see Osma & Farchione [14]), indicated that 65–75% of participants showed a reduction in anxiety and depressive scores in the post-assessment, and most of them maintained these improvements at the 6-month follow-up. Our results also revealed that some participants improved in anxiety and depressive scores but only at the 6-month follow-up. While recent efforts have been carried out to implement the UP in a reduced number of sessions (for instance, De Paul & Caver [57] or Socias-Soler et al. [58]) that resulted in more cost-effective solutions to address psychological disorders in adults, results derived from this work may suggest that, in chronic health conditions, longer programs are needed to train some UP modules more extensively (i.e., cognitive flexibility, understanding of physical sensations or emotional exposure) and to achieve the desired changes in anxiety and depressive scores.

It has been suggested that emotion regulation skills are associated with physical and psychological health in populations with chronic physical diseases [59]. For this reason, an additional outcome that was assessed in our study was emotion dysregulation. In our study, seven participants had high emotion dysregulation at pre-assessment; only four of them showed improvements at post-assessment and only two participants maintained those improvements by the 6-month follow-up. Long COVID-19 patients have to cope with multiple challenges [18,19] that are rarely solved in eight weeks of treatment, not only because participants may need more time to train emotion regulation skills, but also because some procedures are out of their control and are generally solved in the long term (i.e., going through medical tribunals to determine if patients should return to work, if their work has been adapted or if they should be given a permanent incapacity for work). Future studies could explore whether regular follow-up sessions with the therapist after the intervention ends help participants recall and apply these skills during particularly challenging situations.

Another contribution of this study is to explore the psychopathological profile of long COVID-19 patients and to analyze changes in MEDI scores after the implementation of the UP. Our results indicated that, although participants presented different clinical diagnoses at pre-assessment, not all of them had clinical symptoms in emotional disorder dimensions measured with the MEDI. The most impaired dimensions at pre-assessment were positive temperament and depressed mood but scores in these dimensions had improved in very few participants at post-assessment and follow-up. The remaining dimensions had also improved in few participants at post-assessment and at follow-up. The small size of our sample prevented from drawing robust conclusions about the possible impact of the UP in the improvement of the psychopathological profile of long COVID-19 patients but, it is possible that changes in some dimensions of MEDI in chronic conditions such as long COVID-19 may require longer interventions. While merely speculative, it is also possible that more noticeable changes occurred when scores in some psychological dimensions were higher at pre-assessment. It was recently found that, after the implementation of the UP, larger improvements in neuroticism and negative affect occurred when participants showed increased scores on those variables at the baseline assessment point [60].

Recent studies revealed that quality of life is extremely impaired in long COVID-19 patients because they have to completely change their lifestyle, including normal activities, engaging in family and social relationships and employment [45,61,62]. This appears to be supported by our findings, as almost all participants had low scores in quality of life at pre-assessment. Similarly to past studies [56], encouraging results were found after implementing the UP, which translated in the improvement of quality of life scores for seven participants. Unfortunately, not all participants had maintained these improvements by the 6-month follow-up. It has been suggested that breathlessness and fatigue are two of the biggest contributors to decreased quality of life in long COVID-19 patients [61]. In our study, we assessed long COVID-19 physical symptoms only at pre-assessment and we did not formally assess them longitudinally so we cannot determine its association with improvement in quality of life at post-assessment and follow-ups. Therefore, future studies should explore whether changes in quality of life can be attributed to the psychological intervention itself or whether they are be better explained by improvements in the physical burden of long COVID-19.

While this study contributes to the existing literature regarding the preliminary feasibility and the clinical utility of the UP for the psychological management of emotional disorders in long COVID-19 populations, limitations of this study should also be acknowledged. First, due to the characteristics of the ARACOV general project, sample size obtained in our study was lower than expected. This prevents us from conducting the proposed multiple baseline study and from extracting more robust conclusions about the clinical relevance of the UP in long COVID-19 patients. We hope that in the near future we will be able to continue recruiting participants and can conduct the multiple baseline study that will serve to confirm some of the findings presented in this pilot study and propose a more robust randomized controlled trial with a control group. A recent cohort study with COVID-19 patients suggested that improvement in depressive symptoms among individuals with long COVID-19 may be related to the physiological recovery from long COVID-19 symptoms (i.e., reduction in inflammatory levels) [21]. This study also found that, although some participants with long COVID-19 experience relief in their depressive symptoms, most participants continued reporting higher levels of depressive symptoms in the two years following infection compared to pre-infection levels [21]. In our study the absence of a control group prevents us from determining whether spontaneous remission of anxiety and depressive scores may have occurred. Therefore, this represents a potential line of research for future controlled studies that include a comparison group.

Also related to the characteristics of the ARACOV project, participants in our study were those who did not met the eligibility criteria for the study implementing the nutritional and rehabilitation intervention. This inclusion criteria may have affected the representativeness of our sample, potentially limiting the generalizability of the findings. Specifically, our sample may differ from the broader population of patients with long COVID, which should be considered when interpreting the results. It is also worth noting that, due to this limitation in participant recruitment, only 12 patients with long COVID were included in the UP psychological intervention. The limited sample size constrains both the generalizability of the findings and the possibility of drawing more definitive conclusions regarding the clinical utility of the UP psychological intervention. Therefore, the results presented in this study should be interpreted with caution. An additional limitation of our study is the high dropout rates found at the 6-month follow-up in the assessment protocol. To reduce the burden of assessment protocols, we recommend future studies select the most important variables to be assessed over the course of treatment. In our case, we believe that some variables that helped us to better understand the results derived from our study (i.e., the presence and evolution of long COVID-19 physical symptoms and practice of emotion regulation abilities across all assessment points) should be formally asked during the UP program and at follow-up sessions. Additionally, we did not ask participants about the specific lifestyle and physical activity recommendations they had received from their healthcare providers, which could have provided valuable context for interpreting their self-reported healthy behaviors (i.e., alcohol consumption and practice of exercise). Finally, it is also possible that other relevant symptoms that are present in long COVID-19 patients (attention and memory difficulties, mental fog, distractibility, etc.) hinder the ability to answer the questionnaires. This could be reduced by conducting psychosocial assessments using technologies which allow to change font size and color, reply by simple ticking the answer or even answer by sending audios instead of written responses.

Despite these limitations, to the best of our knowledge, this is the first study that explores the preliminary feasibility and clinical utility of the UP for long COVID-19 patients. The transdiagnostic approach of this intervention and the well-accepted online format used in this study encourage us to suggest that providing psychological care to patients suffering a silenced medical condition with comorbid emotional disorders is feasible. Five years after the pandemic, most survivors have resumed their lives as they were before the pandemic. Unfortunately, long COVID-19 patients have seen their usual lives come to a stop and they do not know if they will continue again at some point. Additionally, long COVID-19 patients rarely receive the psychological care they need, leaving them feeling ignored and desperate for support [45]. Our promising results for alleviating emotional disorders in long COVID-19 patients encourage us to propose a future randomized controlled trial to help us confirm the efficacy of this transdiagnostic psychological intervention to reduce the emotional burden faced by long COVID-19 populations.

## Supporting information

S1 File English-approved protocol PU Aracov V3. (DOCX)

S2 File Spanish-approved protocol PU Aracov V3. (DOCX)

S1 Checklist Trendstatement trend checklist. (PDF)
