# Supplementary material for: Preliminary feasibility and clinical utility of the Unified Protocol for the transdiagnostic treatment of emotional disorders in people with long COVID-19 condition: A single case pilot study
Source: PLoS One. 2025 Aug 8;20(8):e0329595. doi: 10.1371/journal.pone.0329595 (PMC12334038; doi:10.1371/journal.pone.0329595)
Supplement: S1 File — (DOCX) [file pone.0329595.s001.docx]

# STUDY PROTOCOL

## Study code: V3.0_15_11_22

**Version number and date:** Version 3.0 of 15-11-2022.

**Study title:** Efficacy of the Unified Protocol for the improvement of emotional symptomatology and/or Emotional Disorders in people with Long Covid.

## Research Team:

- **Principal Investigator:** Jorge J. Osma López.
- **Interlocutors/coordinators in the center:** Jorge J. Osma López.
- **Service or Unit:** Health Research Institute of Aragon (IISA)

**Sponsor:** Health Research Institute of Aragon (IISA)

**Founder:** Solutex GC S.L.

# INDEX

1. Background and rationale for the study
   1. When COVID-19 Symptoms Persist: The Post COVID Condition (Long Covid)
   2. Emotional symptomatology in patients with Long Covid
   3. Transdiagnostic interventions based on emotional regulation
2. Objectives
3. Hypothesis
4. Methodology:
   1. Study population
   2. Selection criteria
   3. Sample size calculation
   4. Instruments
   5. Statistical analysis
   6. Interventions to be carried out
5. Ethical aspects
6. Schedule
7. Budget
8. Bibliography

# INTRODUCTION/JUSTIFICATION OF THE STUDY

## When COVID-19 Symptoms Persist: The Post COVID Condition (Long Covid)

The COVID-19 disease, which originated in Wuhan (China) in December 2019, has caused one of the largest pandemics in world history. Two years after the WHO declared COVID-19 pandemic status, the disease has spread to 220 countries and infected more than 469 million people, with more than 6 million deaths (WHO, 2022). In Aragon, to date, approximately 401,000 cases have been reported, of which 4917 have died (Government of Aragon, 2022).

A proportion of patients with Covid-19 do not manage to recover their initial state of health and report persistent symptoms over time. The number of people affected with long-term sequelae after COVID-19 is unknown, but published reports indicate that it could be approximately 10-20% of patients. This group of people suffers from what has been defined by the WHO as a post-COVID-19 condition (Long Covid). Thus, those people with a probable or confirmed history of SARS CoV 2 infection, who present symptoms, which cannot be explained by other causes, three months after the onset of COVID-19 or at least two months, would suffer from Long Covid. Studies have shown that Long Covid can affect the entire spectrum of Covid-19 patients, from those with very mild acute illness to the most severe forms (WHO, 2021). However, a higher incidence of persistent COVID-19 symptoms has been observed in patients with comorbidities or more severe infection (Mandal et al., 2021).

Regarding the data in Aragon, these indicate that 10% of patients diagnosed with Covid-19 since the beginning of the pandemic could suffer from Long Covid, which means a total of approximately 40000 patients (Government of Aragon, 2022).

Among this group of patients with COVID-PC, the most common symptoms observed are fatigue, shortness of breath and cognitive dysfunction, but there may also be other symptoms that usually have an impact on the patient's daily functioning (WHO, 2021). Thus, more specifically, within this condition, a large number of symptoms have been reported that affect different systems: neurocognitive (brain fog, dizziness, loss of attention, confusion), autonomic (chest pain, chest pain, tachycardia, palpitations), gastrointestinal (diarrhea, abdominal pain, vomiting), respiratory (general fatigue, dyspnea, cough, sore throat), musculoskeletal (myalgia, arthralgias), psychological (post-traumatic stress, anxiety, depression, insomnia), and other manifestations (ageusia, anosmia, parosmia, skin rashes) (Fernández-de-Las-Peñas et. al, 2021).

## Emotional symptomatology in patients with Long Covid

The pandemic situation caused by COVID-19 and the measures adopted, such as confinement or the reduction of social contacts, have had a great emotional impact on the entire population, with a higher prevalence of psychological sequelae observed among those who have suffered from the disease (Mazza et al., 2020; Khraisat et al., 2021). A meta-analysis that included 9605 people who had suffered from COVID-19, reports alarmingly high rates of persistent psychological distress (36%), anxiety disorders (22%), depression (21%), post-traumatic stress disorder (20%), and sleep disorders (35%) (Khraisat et al., 2021) in this

population. Thus, the psychological sequelae present in those with Long Covid could be framed within the group of Emotional Disorders (TEs), a nomenclature that groups anxiety, depressive and related disorders (Barlow et al., 2018).

As for the risk factors related to these psychological symptoms in people with Long Covid, some have been observed, such as being a woman, having a history of psychiatric disorders, the severity of the disease, having medical comorbidities, or having little social support (Thye et al., 2022). Thus, the exact cause of these psychological sequelae is yet to be determined, as it could be the direct action of the coronavirus on the brain and Central Nervous System, indirect effects through systemic responses to the virus, or the result of psychological stressors (Thye et al., 2022). Some of the latter are uncertainty, concern about the future, fear regarding infection, economic or social isolation, and the experience of being in the ICU (Thye et al., 2022; Leviner, 2021).

In addition to the psychological sequelae mentioned above, there is diagnostic uncertainty due to the multifactorial nature of Long Covid and the stigma related to symptom skepticism, aspects that affect the functional capacity, social and family life, work capacity, and quality of life of people with COVID-19 (Brown and O'Brien, 2021; ONS, 2021).

What has been discussed in previous paragraphs suggests that there are great mental health needs to be met among people with Long Covid, who should be considered a risk group. Thus, it is necessary to apply intervention programs for the treatment of the psychological sequelae derived from Long Covid,

as well as the emotional demands and needs in this population highly affected by the COVID-19 pandemic, with emotional disorders being the most prevalent in it.

## Transdiagnostic interventions based on emotional regulation

Emotional regulation (ER) is defined as "those processes by which people exert an influence on the emotions we have, on when we have them, and on how we experience and express them" (Gross, 1999, p. 275). Emotional dysregulation, understood as the failed attempt to regulate emotions in an adaptive way, is at the basis of EDs (Gratz, 2003; Linehan, 1993). The growing literature on emotional regulation (ER) has shown that people with EDs show difficulties when using ER strategies, making maladaptive use of them, and thus contributing to the maintenance of symptoms (Osma et al., 2021).

In recent years, Cognitive Behavioral Therapy (CBT) has proposed interventions with a transdiagnostic approach, that is, focused on the shared underlying mechanisms of EDs (McManus et al., 2010). As we have mentioned in the previous paragraph, this group of disorders presents problems in ER. In turn, it shares a series of etiological and maintenance mechanisms, such as high neuroticism (Brown & Barlow, 2009). In this regard, several studies suggest that high dimensions of this personality dimension are directly associated with different medical disorders (Smith & MacKenzie, 2006; Suls et al., 2005), indicating that it could play a key role in the relationship between Long Covid and its main psychological symptoms.

The Unified Protocol for the Transdiagnostic Treatment of Emotional Disorders (UP; Barlow et al., 2018) is an example of a transdiagnostic and cognitive-behavioral intervention explicitly aimed at treating high neuroticism, or in other words, training in adaptive ER skills (Barlow et al., 2018). By focusing on the mechanisms common in the different EDs, UP offers numerous advantages over protocols designed for the treatment of specific disorders, such as allowing the approach of people with comorbidity (Brown et al., 2001).

Regarding its usefulness, UP has shown efficacy in reducing symptoms of anxiety and depression, neuroticism, disorder-specific symptoms and diagnostic criteria and in increasing extraversion, quality of life and emotional regulation skills in people with EDs, with and without comorbidity (Cassiello-Robbins et al., 2020; Leonardo et al., 2021; Sakiris & Berle, 2019). In Spain, our research team has carried out the first randomized multicenter clinical trial applying UP in a group format in patients diagnosed with ED in mental health units of our National Health System (Osma et al., 2018), finding similar results (Osma, Peris-Baquero, Quilez-Orden, et al., 2021; Osma, Peris- Baquero, Suso-Ribera, et al., 2021). In turn, a recent systematic review reports on the preliminary usefulness of UP for the treatment of emotional symptomatology in a population that also has a medical condition, in turn producing improvements in medical symptomatology in some cases (Osma et al. 2021).

For all of the above, and given that the prevalence of emotional symptoms and EDs in people with Long Covid is high, the application of UP could be very useful in their treatment. In this way,

our starting hypothesis is that a structured psychological intervention focused on treating emotional dysregulation (UP) will generate an improvement in the emotional state of this group of patients.

# OBJECTIVES OF THE STUDY

## General objective:

To study the efficacy and acceptability of the "Unified Protocol for the transdiagnostic treatment of emotional disorders" in the treatment of emotional symptomatology and/or EDs in a sample of patients with Long Covid.

## Specific objectives:

1. To contribute to the advancement of knowledge about the treatment of emotional symptoms and/or EDs present in people with Long Covid.
2. To study the efficacy of UP for the treatment of emotional symptomatology and/or EDs over time through follow-ups at one month, and at 3, 6 and 12 months after the end of the intervention.
3. To evaluate adherence to the program, acceptability and improvement in measures after the application of UP in an online individual format in a sample of people with Long Covid.

# TESTING OF HYPOTHESES

## General hypothesis:

UP will be shown to be effective for the treatment of emotional symptoms and/or EDs in a sample of patients with Long Covid.

## Specific hypotheses:

H1. Statistically significant improvements, with moderate effect sizes, will be obtained after the intervention in all measures used.

H2. The improvements obtained after the application of the UP will be maintained in the medium and long term, in the follow-ups at one month and at 3, 6 and 12 months.

H3. Participants will report high acceptability and satisfaction scores regarding the intervention, its components, and the application format.

# METHODOLOGY

## Study population

The population that will be included in the study will be made up of members of the cohort constituted in the ARACoV 1 project (CEICA approval act No. 14/2022) who have not been included in the trial "Specialized nutritional intervention and rehabilitative treatment to improve quality of life in a cohort of patients with post-covid-19 condition". Thus, the participants will be adults, belonging to the autonomous community of Aragon, with clinical criteria of having suffered from COVID-19 with a diagnosis confirmed by diagnostic techniques and systems (PCR, rapid antigen tests, serological test for the detection of antibodies) and who remain with symptoms beyond 12 weeks after the onset of symptoms.

## Selection criteria

The inclusion criteria for participation in the project are: (1) Belong to the autonomous community of Aragon (2) Be at least 18 years old, (3) Have a good understanding of Spanish (4) SARS CoV 2 infection documented by PCR, Ag test or serology (positive anti-N A) (5) Persistence of symptoms beyond 12 weeks after acute SARS CoV 2 infection, (6) Present a score equal to or greater than 8 points in anxious symptomatology (OASIS) and/or equal to or greater than 7 points in depressive symptomatology (ODSIS) (7) Have access to the Internet, (8) Sign the informed consent.

The exclusion criteria are: (1) Participate in the trial "Specialized nutritional intervention and rehabilitative treatment to improve quality of life in a cohort of patients with Long Covid", (2) Psychological symptoms already existed before acute SARS CoV infection, (3) Be receiving psychological and/or pharmacological treatment for a mental disorder at present, (4) Have a diagnosis of severe mental disorder, (5) Active suicidal ideation at the time of evaluation.

## Sample size calculation

We will conduct a single-case experimental design with multiple baseline to avoid the need for a control group (in case designs the baseline is used as a control for each participant). Current guidelines (Kratochwill et al., 2010; Kratochwill et al., 2012) recommend 3 different baselines, so participants will be assigned to one of these 3 conditions: initiation of the intervention after 6 days of assessment (condition 1), after 8 days of assessment (condition 2) or after 10 days of assessment (condition 3). To calculate the sample size of this pilot study, we relied on a study that proposed rules of thumb for pilot studies based on the size of the desired effect and the size of a subsequent main trial (Bell et al., 2018). Thus, to find an effect size of up to 0.3, with a power of 80% and assuming that study 2 will have a sample of 130 participants, the study by Bell et al. (2018) states that the sample size of the pilot study should be 20 participants per condition, in this case, as it has three different baselines, the sample size will be 60 participants.

## Instruments

1. **Sociodemographic data and medical history:** sex, age, place of residence, marital status, employment status, health habits, SARS CoV 2 vaccination.

## Evaluation protocol:

- - *Table of COVID-19 symptoms reported by the patient:* To facilitate the collection of the symptoms reported by the patient regarding Long Covid, we have developed a table in which different symptoms present in this condition are collected, grouped into the following categories: General Symptoms, Respiratory Symptoms, Gastrointestinal Symptoms, Musculoskeletal Symptoms, Cutaneous Symptoms, Otolaryngological Symptoms, Neurological Symptoms, Cardiovascular Symptoms and Others.
  - *Structured interview for anxiety disorders and related disorders, according to the DSM-5 (ADIS-5; Brown & Barlow, 2014):* Structured interview that follows the DSM-5 criteria for anxiety, mood, and related disorders. Adjustment disorders will be assessed according to DSM-5 criteria.
  - *General Depression Severity and Interference Scale (ODSIS; Bentley et al., 2014; Osma et al., 2019):* Evaluation through 5 items of the frequency, intensity, severity and interference of depressive symptomatology.
  - *General Anxiety Severity and Interference Scale (OASIS; Norman et al., 2006; Osma et al., 2019):* Evaluation through 5 items of the frequency, intensity, severity and interference of anxious symptomatology.
  - *EuroQol-5D (Brooks, 1996. Validada al castellano por Badia et al., 1999):*

Assessment of self-perceived health status.

- - *Multidimensional Inventory for Emotional Disorders (MEDI; Rosellini & Brown, 2019; Osma et al., 2022):* Evaluation through 49 items of the transdiagnostic profile of Emotional Disorders, which is composed of nine dimensions: neurotic temperament, positive temperament, depressed mood, somatic anxiety, arousal activation, social anxiety, intrusive cognitions, traumatic re-experience, and avoidance.
  - *Emotional Regulation Difficulties Scale (DERS; Gratz & Roemer, 2004. Validated in Spanish by Hervás & Jódar, 2008):* Evaluation through 28 items of difficulties in emotional regulation through 5 subscales: lack of control, rejection, interference, inattention, and emotional confusion.
  - *Stress Tolerance Scale (DTS; Simons & Gaher, 2005. Validated in Spanish by Sandin et al., 2017):* Evaluation through 15 items of tolerance to discomfort. It assesses the following dimensions: 1) Perceived ability to tolerate emotional distress; 2) Subjective assessment of discomfort; 3) Attention absorbed by negative emotions; 4) Regulatory efforts to alleviate discomfort.
  - *Treatment Satisfaction Questionnaire (STQ; adapted from the Client Satisfaction Questionnaire [CSQ-8] by Larsen et al., 1979):* Our adaptation includes 6 of the 8 items of the CSQ-8 (perceived quality, adequacy to the

previous expectations, recommendation of treatment to friends or relatives, usefulness of the techniques learned, general satisfaction with the intervention and probability that they will choose an intervention of this type again) and one more item related to the discomfort generated by the intervention. Likewise, a change has been made in the response Likert scale, going from 4 points in the original (0= "Bad/Nothing" to 4= "Excellent/Great") to 11 in the current one (0= "Bad/Not at all to 10= "Excellent/Very much"). In addition, the following 5 questions were added in an open format: *Is there any other content that you think would be interesting to include in the program?; Do you think there is any content in the program that does not need to be addressed?; The duration of the program, 5 sessions of 2 hours, do you think it is enough? What is your opinion about the type of format used to apply the program? Here is a space for you to express any questions about the program.*

- - *Evaluation questionnaire for the modules of the Unified Protocol (UP):* Questionnaire prepared *ad hoc* composed of 7 questions; one of a general nature that evaluates the usefulness of the program to improve emotional regulation and six specific questions that separately evaluate the usefulness to better regulate emotions of each of the techniques that are worked on in the different modules of the UP. The response scale is Likert type and ranges from 0 (not at all) to 10 (a lot).
  - *Baseline Assessment Questionnaire: For* the daily baseline assessment, we will use the ODSIS questionnaires (Bentley et al., 2014; Osma et al., 2019) and OASIS (Norman et al., 2006; Osma et al., 2019) modifying the temporality to which the questions refer; going from "during the last week" in the original questionnaires to "during the day yesterday" in the version used for the evaluation of the baseline.

## Statistical analysis

The analyses will be carried out using the IBM SPSS Statistics version 22.0 statistical package for Windows (IBM Corp., 2013). First, normality tests will be carried out to check whether or not the sample follows a normal distribution. Next, descriptive statistical analyses will be carried out with the aim of

objective of obtaining an overview of the scores in the variables and sociodemographic data. Parametric or non-parametric analyses will be carried out (depending on whether or not the sample follows a normal distribution). First, analyses of variance (ANOVA) will be carried out in order to see if there are differences between the three conditions at the different evaluation times. Secondly, repeated measures ANOVA analyses will be carried out to analyse the evolution in each of the conditions. In the event that the sample does not follow a normal distribution, equivalent non-parametric tests will be carried out with the same objective. For all statistical analyses, effect sizes will be calculated using Cohen's d statistic, whose estimates are usually interpreted as small (d ≈ 0.2), medium (d ≈ 0.5), or large (d ≈ 0.8). Another aspect that we consider important to analyze is how participants change their scores on the ODSIS and OASIS scales depending on the content addressed in each module. To this end, a visual analysis of the changes in the scores will be carried out through the statistical software R (version 4.1.0; R Core Team, 2021), to see how the slopes change in the different phases of the study (evaluation and treatment), and in the different modules within the treatment. To carry out this visual analysis, the responses of the ODSIS and OASIS scales will be used, which the participants will fill in at the pre-program moments, weekly during the program, post-program and in the follow-ups at one month and 3 months. Finally, qualitative analyses, specifically content analysis, will be carried out using the statistical software MAXQDA (Kuckartz and Rädiker, 2019) to analyse the answers to the open questions on the assessment of the satisfaction of the programme, its contents and the format.

## Interventions to be carried out

- - 1. **Recruitment and dissemination of the study**

The recruitment of participants will be carried out through the cohort constituted in the ARACoV 1 project (CEICA approval act No. 14/2022). From the ARACoV 1 project, information will be provided about the study and the possibility of participating will be offered to those people belonging to the ARACoV 1 cohort who have not been included in the trial "Specialized nutritional intervention and rehabilitative treatment to improve quality of life in a cohort of patients with post-covid-19 condition" belonging to said project and who have a score equal to or greater than 8 points in anxious symptomatology (OASIS) and/or equal to or greater than 7 points in depressive symptomatology (ODSIS), having completed these scales within the ARACoV 1 project and after signing the informed consent of said project. Those interested in participating will receive the information sheet about the study and informed consent from the nurse member of the ARACoV 1 project team in charge of recruiting the ARACoV 1 cohort who has previously informed them about the study. After having accessed all the information about the study contained in the information and informed consent sheet, those who wish to participate may sign the informed consent form on the study and the LOPD.

With those who agree to participate, an individual online session will be arranged with the psychologist in order to assess the possible emotional disorder of the patient. In this session it will also be assessed the presence of a serious mental disorder and/or active suicidal ideation, the latter two being exclusion criteria. If after the evaluation exclusion criteria are detected the patient will be informed that they cannot continue to participate in the study and the participant will be advised to seek professional help and be guided to do so. After the evaluation, the participant will be sent a Google Forms link through which they will respond to the pre-intervention evaluation protocol.

Once they have completed it, they will be informed that in the coming weeks they will receive an email notifying them of the condition to which they have been randomly assigned: start of the intervention after 6 days of evaluation (condition 1; 6 days of baseline), after 8 days of evaluation (condition 2; 8 days of baseline) or after 10 days of evaluation (condition 3; 10 days of line base). Randomization to the different baselines will be performed with the randomization software (www.randomizer.org). In the program, the researcher will generate a set of 60 numbers.

## Development of the intervention

The fundamental idea of choosing this type of design is that each of the participants can be their own control (Kratochwill et al., 2013). Thus, as explained above, participants will be randomized to one of the 3 conditions, 6, 8 or 10 days of baseline. Each condition will be made up of 20 participants.

The intervention program based on the UP will be applied for 8 sessions of 1 hour, in online format through the Google Meet platform. During the sessions, the exercises of the previous session will be corrected (except in session 1), the new contents of the session will be introduced, a V/F survey will be carried out to ensure that they have understood the key concepts of the session, exercises will be carried out in session and the practice of the exercises between sessions will be encouraged.

Participants will receive a brief manual containing the important contents of each session, the exercises to be carried out and the corresponding records. The contents of the sessions will be:

| **Title** | **Content/Emotional Regulation Skill** | **Practice** |
| --- | --- | --- |
| Session 1. Goal setting and maintaining motivation | Setting Treatment Goals  Decisional Balance Exercise | Define the overall treatment goals, as well as the steps to achieve them. Explore the pros and cons of changing and staying the same, reflecting on it to tip the balance in favor of change. |
| Session 2. Understanding Emotions | Functional Analysis of Emotional Response | Analyze the emotional response through the ARCO record and assess the short and long consequences  of our emotional responses. |
| Session 3. Mindful emotion awareness | Observe what is happening in the present without judgment | Practice of different meditation exercises |
| Session 4. Cognitive Flexibility | Observing our thoughts and opening up the range of possibilities of interpretation | Observe the thoughts we have and find out if they reflect reality and bring us closer to our goals and, if not, train in |

|  | Troubleshooting Technique | generate new interpretations of the situation that are more  realistic and useful/or apply problem solving. |
| --- | --- | --- |
| Session 5. Countering Emotional Behaviors | Identify the emotional behaviors we perform and describe opposite or alternative behaviors | We will identify the emotional behaviors we use, assess their usefulness, describe alternative or opposite behaviors and assess their usefulness. |
| Session 6. Interoceptive Exposures | Gradual exposure to internal stimuli that generate intense discomfort in order to tolerate the discomfort progressively | On the one hand, we will design and carry out exercises that provoke the physical sensations present in emotions and we will tolerate them little by little until they no longer have a negative effect on our behavior. |
| Session 7. Emotional Exposures | Gradual exposure to internal and/or external stimuli that generate intense discomfort in order to tolerate the discomfort progressively | We will elaborate a hierarchy of exposure to different emotions in order to progressively expose ourselves to each of them and thus tolerate them little by little until they no longer have a negative effect on our  behavior. |
| Session 8. Relapse prevention | Review of skills learned and progress during the intervention.  Practice plan after the intervention.  Record of difficult situations and how to deal with them. | We will review the progress to have an overview of everything we have learned and be aware of the skills we need to continue training.  We will identify difficult risk situations so that we can recognize them in time and thus know what we have skills to face them and make sure that we have practiced them. |

Once the intervention is finished, particiants will be send the Google Forms link by email to fill in the post-program protocol. Follow-ups will be carried out after the end of the program at one month and after 3, 6 and 12 months following the same procedure as the post-program assessment.

# ETHICAL ASPECTS

All the people who are evaluated and meet the inclusion criteria to participate in the trial will sign the Personal Data Protection document so that they have an idea of who is going to use the results of this research and for what purpose.

Participants who are going to receive intervention will be informed about the treatment procedures, as well as its duration and the phases of the study. To this end, they will be sent an information sheet explaining the treatment, and its respective phases, as well as the document corresponding to the Informed Consent.

With regard to the confidentiality, processing, communication and transfer of the personal data of all participating subjects, it will be in accordance with the provisions of the Declaration of Helsinki (Seoul, 2008), Law 14/2007 on Biomedical Research, Organic Law 3/2018, of 5 December, on the Protection of Personal Data and the Guarantee of Digital Rights (LOPD GDD). As of May 25, 2018, the new legislation on personal data in the EU is fully applicable, specifically Regulation (EU) 2016/679 of the European Parliament and of the Council of April 27, 2016 on Data Protection (GDPR). In accordance with the provisions of the aforementioned legislation, participants may exercise their rights of access, modification, opposition and cancellation of data, for which they must contact the principal investigator in charge of the study. The personal information collected for the study will be replaced by alphanumeric codes and the sociodemographic data will be stored separately and will only be accessible by the researchers responsible for the storage and processing of the data, always protecting the right to privacy.

The information will be collected through the Google Forms application, which is included in Unizar's "Google Workspace for Education" package. Through this package, Google offers email, documents, and storage space to the University of Zaragoza. This package of programs is offered in cloud mode and, therefore, the characteristics and linkage in relation to the applicable regulations in Spain must be taken into account, which to be considered initially would be the National Security Scheme and the current regulation on Data Protection and Guarantee of Digital Rights. In this sense, as the manufacturer itself points out, Google's Cloud solutions have obtained compliance for the National Security Scheme "HIGH Level".

On the other hand, and in accordance with the regulations for the protection of Personal Data, there is a commitment and declaration by Google to comply with them. Likewise, the services offered by Google are limited to the requirements regulated by European Regulation 2016/679 (GDPR), within the agreement on data protection of third countries with the European Union. Google Workspace for Education is governed by the following standards and certifications:

ISO/IEC 27001 (Information Security Management) ISO/IEC 27017 (Cloud Security)

ISO/IEC 27018 (Cloud Privacy)

ISO/IEC 27701 (Management of Privacy Information)

# SCHEDULE

Recruitment of participants is scheduled to begin in October 2022 and run until June 2023.

# BUDGET

- Full-time senior research technician contract to carry out the interventions (contract start 3 October 2022 to 3 October 2023). The contract runs to ARACoV 1 project managed by the Health Research Institute of Aragon.

# BIBLIOGRAPHY

Barlow, D. H., Farchione, T. J., Sauer-Zavala, S., Latin, H. M., Ellard, K. K., Bullis, J. R., Bentley, K., Boettcher, H., y Cassiello-Robbins, C. (2018). *Unified protocol for transdiagnostic treatment of emotional disorders: Therapist guide (2nd ed.).* New York, NY: Oxford University Press.

Bell, M. L., Whitehead, A. L., & Julious, S. A. (2018). Guidance for using pilot studies to inform the design of intervention trials with continuous outcomes. *Clinical epidemiology, 10,* 153–157. https://doi.org/10.2147/CLEP.S146397

Bentley, K., Gallagher, M., y Barlow, D. (2014). Development and validation of the Overall Depression Severity and Impairment Scale. *Psychological Assessment*, *26*(3), 815–830. https://doi.org/10.1037/a0036216

Brown, T. A., y Barlow, D. H. (2009). A proposal for a dimensional classification system based on the shared features of the DSM-IV anxiety and mood disorders: Implications for assessment and treatment. *Psychological Assessment*, *21*(3), 256. <https://doi.org/10.1037/a0016608>

Brown, T. A., & Barlow, D. H. (2014). Anxiety and related disorders interview schedule for DSM-5 (ADIS-5)-adult and lifetime version: Clinician manual. Oxford University Press.

Brown, T. A., Campbell, L. A., Lehman, C. L., Grisham, J. R., y Mancill, R. B. (2001). Current and lifetime comorbidity of the DSM-IV anxiety and mood disorders in a large clinical sample. *Journal of Abnormal Psychology*, *110*(4), 585. [https://doi.org/10.1037//0021-843x.110.4.585](https://doi.org/10.1037/0021-843x.110.4.585)

Brown, D. A., & O’Brien, K. K. (2021). Conceptualising Long COVID as an episodic health condition. *BMJ Global Health*, *6*(9), e007004.

Cassiello-Robbins, C., Southward, M. W., Tirpak, J. W., y Sauer-Zavala, S. (2020). A systematic review of Unifed Protocol applications with adult populations: Facilitating widespread dissemination via adaptability. *Clinical Psychology Review, 78,* 101852.

Fernández-de-Las-Peñas, C., Palacios-Ceña, D., Gómez-Mayordomo, V., Cuadrado, M. L., & Florencio, L. L. (2021). Defining post-COVID symptoms (post-acute COVID, long COVID, persistent post-COVID): an integrative classification. *International journal of environmental research and public health*, *18*(5), 2621.

Government of Aragon (2022). Covid 19. Current situation. <https://transparencia.aragon.es/COVID19>

Gratz, K. L. (2003). Risk factors for and functions of deliberate self-harm: An empirical and conceptual review. *Clinical Psychology: Science and Practice*, *10*(2), 192.

Gratz, K. L., y Roemer, L. (2004). Multidimensional assessment of emotion regulation and dysregulation: Development, factor structure, and initial validation of the difficulties in emotion regulation scale. *Journal of psychopathology and behavioral assessment*, *26*(1), 41-54.

Gross, J. J. (1999). Emotion regulation: Past, present, future. *Cognition & emotion*, *13*(5), 551-573.

Hervás, G., & Jódar, R. (2008). Adaptation to Spanish of the Scale of Difficulties in Emotional Regulation. *Clínica y Salud*, *19*(2), 139–156.

Khraisat, B., Toubasi, A., AlZoubi, L., Al-Sayegh, T., & Mansour, A. (2021). Meta- analysis of prevalence: the psychological sequelae among COVID-19 survivors. *International Journal of Psychiatry in Clinical Practice*, 1-10

Kratochwill, T. R., Hitchcock, J., Horner, R. H., Levin, J. R., Odom, S. L., Rindskopf,

D. M., & Shadish, W. R. (2010). Single-case designs technical documentation. What works clearinghouse.

Kratochwill, T. R., Hitchcock, J. H., Horner, R. H., Levin, J. R., Odom, S. L., Rindskopf, D. M., & Shadish, W. R. (2013). Single-case intervention research design standards. *Remedial and Special Education*, *34*(1), 26-38.

Larsen, D. L., Atkinson, C. C., Hargreaves, W. A., y Nguyen, T. D. (1979). Assessment of client/patient satisfaction: Development of a general scale. *Evaluation and Program Planning, 2*, 197-207.

Leonardo, C., Aristide, S., y Michela, B. (2021). On the efficacy of the Barlow Unified Protocol for Transdiagnostic Treatment of Emotional Disorders: A systematic review and meta-analysis. *Clinical Psychology Review*, 101999.

Leviner, S. (2021). Recognizing the clinical sequelae of COVID-19 in adults: COVID- 19 Long-Haulers. *The Journal for Nurse Practitioners*, *17*(8), 946-949.

Linehan, M. M. (1993). *Cognitive-behavioral treatment of borderline personality disorder.* Guilford Press.

Mandal, S., Barnett, J., Brill, S. E., Brown, J. S., Denneny, E. K., Hare, S. S., ... & Hurst, J. R. (2021). ‘Long-COVID’: a cross-sectional study of persisting symptoms, biomarker and imaging abnormalities following hospitalisation for COVID-19. *Thorax, 76*(4), 396-398.

Mazza, M. G., De Lorenzo, R., Conte, C., Poletti, S., Vai, B., Bollettini, I., Melloni, E., Furlan, R., Ciceri, F., Rovere-Querini, P., COVID-19 BioB Outpatient Clinic Study group, & Benedetti, F. (2020). Anxiety and depression in COVID-19 survivors: Role of inflammatory and clinical predictors. *Brain, behavior, and immunity*, *89*, 594–600. <https://doi.org/10.1016/j.bbi.2020.07.037>

McManus, F., Shafran, R., y Cooper, Z. (2010). What does a transdiagnostic approach have to offer the treatment of anxiety disorders? *British Journal of Clinical Psychology, 49*(4), 491–505. <https://doi.org/10.1348/014466509X476567>

Norman, S. B., Hami Cissell, S., Means-Christensen, A. J., y Stein, M. B. (2006). Development and validation of an Overall Anxiety Severity And Impairment Scale (OASIS). *Depression and Anxiety*, *23*(4), 245–249. https://doi.org/10.1002/da.20182

Office for National Statistics. (2021). Coronavirus and the social impacts of ‘long COVID’on people’s lives in Great Britain: 7 April to 13 June 2021.

Osma, J., Martínez-Loredo, V., Quilez-Orden, A., Peris-Baquero, Ó., & Suso-Ribera, C. (2021). Validity Evidence of the Multidimensional Emotional Disorders Inventory among Non-Clinical Spanish University Students. *International Journal of Environmental Research and Public Health*, *18*(16), 8251.

Osma, J., Martínez-García, L., Quilez-Orden, A., & Peris-Baquero, Ó. (2021). Unified Protocol for the Transdiagnostic Treatment of Emotional Disorders in Medical Conditions: A Systematic Review. *International journal of environmental research and public health*, *18*(10), 5077.

Osma, J., Peris-Baquero, Ó., Quilez-Orden, A., Suso-Ribera, C., & Crespo, E. (2021). Unified Protocol for the Transdiagnostic Treatment of Emotional Disorders. In E. Fonseca (Ed.), *Manual of Psychological Treatments: Adults* (pp. 195-220). Madrid: Ediciones Pirámide.

Osma, J., Peris-Baquero, O., Suso-Ribera, C., Farchione, T. J., y Barlow, D. H. (2021). Effectiveness of the Unified Protocol for transdiagnostic treatment of emotional disorders in group format in Spain: Results from a randomized controlled trial with 6-months follow-up. *Psychotherapy Research*, 1–14.

Osma, J., Quilez-Orden, A., Suso-Ribera, C., Peris-Baquero, O., Norman, S., Bentley, K., y Sauer-Zavala, S. (2019). Psychometric properties and validation of the Spanish versions of the overall anxiety and depression severity and impairment scales. *Journal of Affective Disorders*, *252*, 9–18. https://doi.org/10.1016/j.jad.2019.03.063

Osma, J., Suso-Ribera, C., Garcia-Palacios, A., Crespo-Delgado, E., Robert-Flor, C., Sanchez-Guerrero, A., ... Torres-Alfosea, M. Á. (2018). Efficacy of the unified protocol for the treatment of emotional disorders in the Spanish public mental health system using a group format: study protocol for a multicenter, randomized, non-inferiority controlled trial. *Health and quality of life outcomes, 16*(1), 1-10.

Rosellini, A. J., y Brown, T. A. (2019). The Multidimensional Emotional Disorder Inventory (MEDI): Assessing transdiagnostic dimensions to validate a profile approach to emotional disorder classification. *Psychological assessment*, *31*(1), 59.

Sakiris, N., y Berle, D. (2019). A systematic review and meta-analysis of the Unified Protocol as a transdiagnostic emotion regulation based intervention. *Clinical psychology review, 72*, 101751. <https://doi.org/10.1016/j.cpr.2019.101751>

Sandín Ferrero, B., Simons, J. S., Valiente García, R. M., Simons, R. M., & Chorot Raso, P. (2017). Psychometric properties of the spanish version of The Distress Tolerance Scale and its relationship with personality and psychopathological symptoms. *Psicothema*.

Simons, J. S., & Gaher, R. M. (2005). The Distress Tolerance Scale: Development and validation of a self-report measure. *Motivation and emotion*, *29*(2), 83-102.

Smith, T. W., y MacKenzie, J. (2006). Personality and risk of physical illness. *Annual Review of Clinical Psychology, 2*, 435–467. <https://doi.org/10.1146/annurev.clinpsy.2.022305.095257>

Suls, J., y Bunde, J. (2005). Anger, anxiety, and depression as risk factors for cardiovascular disease: the problems and implications of overlapping affective dispositions. *Psychological bulletin*, *131*(2), 260–300. [https://doi.org/10.1037/0033-](https://doi.org/10.1037/0033-2909.131.2.260)

[2909.131.2.260](https://doi.org/10.1037/0033-2909.131.2.260)

Thye, A. Y. K., Law, J. W. F., Tan, L. T. H., Pusparajah, P., Ser, H. L., Thurairajasingam, S., ... & Lee, L. H. (2022). Psychological Symptoms in COVID-19 Patients: Insights into Pathophysiology and Risk Factors of Long COVID-

19. *Biology*, *11*(1), 61.

Vilagut, G., Valderas, J. M., Ferrer, M., Garin, O., López-García, E., & Alonso, J. (2008). Interpretation of the SF-36 and SF-12 health questionnaires in Spain: physical and mental components. *Clinical medicine*, *130*(19), 726-735.

Ware Jr, J. E., Kosinski, M., & Keller, S. D. (1996). A 12-Item Short-Form Health Survey: construction of scales and preliminary tests of reliability and validity. *Medical care*, 220-233.

World Health Organization. (2021). A clinical case definition of post COVID-19 condition by a Delphi consensus, 6 October 2021. [https://www.who.int/publications/i/item/WHO-2019-nCoV-Post_COVID-](https://www.who.int/publications/i/item/WHO-2019-nCoV-Post_COVID-19_conditionClinical_case_definition-2021.1) [19_conditionClinical_case_definition-2021.1](https://www.who.int/publications/i/item/WHO-2019-nCoV-Post_COVID-19_conditionClinical_case_definition-2021.1)

World Health Organization. (2022). WHO Coronavirus disease (COVID‐19) dashboard.
