# Supplementary material for: Preliminary feasibility and clinical utility of the Unified Protocol for the transdiagnostic treatment of emotional disorders in people with long COVID-19 condition: A single case pilot study
Source: PLoS One. 2025 Aug 8;20(8):e0329595. doi: 10.1371/journal.pone.0329595 (PMC12334038; doi:10.1371/journal.pone.0329595)
Supplement: S2 File — (DOCX) [file pone.0329595.s002.docx]

# PROTOCOLO DEL ESTUDIO

## Código del estudio: V3.0_15_11_22

**Número y fecha de la versión:** Versión 3.0 del 15-11-2022.

**Título del estudio:** Eficacia del Protocolo Unificado para la mejora de la sintomatología emocional y/o Trastornos Emocionales en personas con CPCOVID.

## Equipo Investigador:

- **Investigador principal:** Jorge J. Osma López.
- **Interlocutores/coordinadores en el centro:** Jorge J. Osma López.
- **Servicio o Unidad:** Instituto de Investigación Sanitaria de Aragón (IISA)

**Promotor:** Instituto de Investigación Sanitaria de Aragón (IISA)

**Financiador:** Solutex GC S.L.

# ÍNDICE

1. Antecedentes y justificación del estudio
   1. Cuando los síntomas por COVID-19 persisten: La condición post COVID (CPCOVID)
   2. Sintomatología emocional en pacientes con CPCOVID
   3. Intervenciones transdiagnósticas basadas en la regulación emocional
2. Objetivos
3. Hipótesis
4. Metodología:
   1. Población de estudio
   2. Criterios de selección
   3. Cálculo del tamaño de la muestra
   4. Instrumentos
   5. Análisis estadístico
   6. Intervenciones a realizar
5. Aspectos éticos
6. Cronograma
7. Presupuesto
8. Bibliografía

# INTRODUCCIÓN/JUSTIFICACIÓN DEL ESTUDIO

## Cuando los síntomas por COVID-19 persisten: La condición post COVID (CPCOVID)

La enfermedad por COVID-19, originada en Wuhan (China) en diciembre de 2019, ha causado una de las mayores pandemias de la historia mundial. Dos años después de que la OMS declarara el estado de pandemia por COVID-19, la enfermedad se ha extendido a 220 países y ha infectado a más de 469 millones de personas, con más de 6 millones de muertes (WHO, 2022). En Aragón, hasta la fecha, se han comunicado aproximadamente 401.000 casos, de los cuales 4917 han fallecido (Gobierno de Aragón, 2022).

Una proporción de los pacientes con Covid-19 no logran recuperar su estado de salud inicial y refieren síntomas persistentes a lo largo del tiempo. Se desconoce el número de personas afectadas con secuelas a largo plazo después de la COVID-19, pero los informes publicados indican que podrían ser aproximadamente el 10-20% de los pacientes. Este grupo de personas padece lo que ha sido definido por la OMS como condición post COVID-19 (CPCOVID). Así, padecerían CPCOVID aquellas personas con historia probable o confirmada de infección por SARS CoV 2, que presentan síntomas, que no pueden ser explicados por otras causas, tres meses después del inicio de la COVID-19 o al menos dos meses. Los estudios han demostrado que la CPCOVID puede afectar a todo el espectro de pacientes con Covid-19, desde aquellos con enfermedad aguda muy leve hasta las formas más graves (OMS, 2021). Sin embargo, se ha observado una mayor incidencia de síntomas persistentes de COVID-19 en pacientes con comorbilidades o infección más grave (Mandal et al., 2021).

Respecto a los datos en Aragón, estos indican que el 10% de los pacientes diagnosticados con Covid-19 desde el inicio de la pandemia podría padecer CPCOVID, lo que supone un total de unos 40000 pacientes aproximadamente (Gobierno de Aragón, 2022).

Entre este grupo de pacientes con CPCOVID, los síntomas observados como más comunes son la fatiga, la dificultad para respirar y la disfunción cognitiva, pero también se pueden dar otros síntomas que suelen repercutir en el funcionamiento cotidiano del paciente (OMS, 2021). Así, más específicamente, dentro de esta condición se ha informado de una gran cantidad de síntomas que afectan a diferentes sistemas: neurocognitivos (niebla cerebral, mareos, pérdida de atención, confusión), autonómicos (dolor de pecho, dolor torácico, taquicardia, palpitaciones), gastrointestinales (diarrea, dolor abdominal, vómitos), respiratorios (fatiga general, disnea, tos, dolor de garganta), musculoesqueléticos (mialgias, artralgias), psicológicos (estrés postraumático, ansiedad, depresión, insomnio), y otras manifestaciones (ageusia, anosmia, parosmia, erupciones cutáneas) (Fernández-de-Las-Peñas et. al, 2021).

## Sintomatología emocional en pacientes con CPCOVID

La situación de pandemia provocada por la COVID-19 y las medidas adoptadas, como el confinamiento o la reducción de contactos sociales han tenido un gran impacto emocional en toda la población, observándose una mayor prevalencia de secuelas psicológicas entre aquellos que han sufrido la enfermedad (Mazza et al., 2020; Khraisat et al., 2021). Un meta-análisis que incluyó a 9605 personas que habían padecido COVID-19, informa de tasas alarmantemente altas de malestar psicológico persistente (36%), trastornos de ansiedad (22%), depresión (21%), trastorno de estrés postraumático (20%) y trastornos del sueño (35%) (Khraisat et al., 2021) en esta

población. Así, las secuelas psicológicas presentes en aquellos con CPCOVID podrían enmarcarse dentro del grupo de Trastornos Emocionales (TEs), nomenclatura que agrupa trastornos de ansiedad, trastornos depresivos y relacionados (Barlow et al., 2018).

En cuanto a los factores de riesgo relacionados con estos síntomas psicológicos en personas con CPCOVID, se han observado algunos como ser mujer, tener antecedentes de trastornos psiquiátricos, la gravedad de la enfermedad, tener comorbilidades médicas o tener poco apoyo social (Thye et al., 2022). Así, la causa exacta de estas secuelas psicológicas está aún por determinar, ya que podría ser la acción directa del coronavirus en el cerebro y el Sistema Nervioso Central, efectos indirectos a través de respuestas sistémicas al virus, o el resultado de factores de estrés psicológico (Thye et al., 2022). Algunos de estos últimos son la incertidumbre, la preocupación por el futuro, el miedo con respecto a la infección, el aislamiento económico o social y la experiencia de estar en la UCI (Thye et al., 2022; Leviner, 2021).

A las secuelas psicológicas nombradas anteriormente, se suman la incertidumbre diagnóstica a causa de la naturaleza multifactorial de la CPCOVID y el estigma relacionado con el escepticismo de los síntomas, aspectos que afectan a la capacidad funcional, la vida social y familiar, la capacidad de trabajo y la calidad de vida de las personas con CPCOVID (Brown y O’Brien, 2021; ONS, 2021).

Lo comentado en párrafos anteriores, sugiere que existen grandes necesidades de salud mental que satisfacer entre las personas con CPCOVID, quienes deben ser considerados un grupo de riesgo. Así, se hace necesario el aplicar programas de intervención para el tratamiento de las secuelas psicológicas derivadas de la CPCOVID,

así como de las demandas y necesidades emocionales en esta población altamente afectada por la pandemia del COVID-19, siendo los trastornos emocionales los más prevalentes en ella.

## Intervenciones transdiagnósticas basadas en la regulación emocional

La regulación emocional (RE) se define como “aquellos procesos por los cuales las personas ejercemos una influencia sobre las emociones que tenemos, sobre cuándo las tenemos, y sobre cómo las experimentamos y las expresamos” (Gross, 1999, p. 275). La desregulación emocional, entendida como el intento fallido de regular las emociones de una forma adaptativa, está en la base de los TEs (Gratz, 2003; Linehan, 1993). La creciente literatura en regulación emocional (RE) ha demostrado que las personas con TEs muestran dificultades a la hora de utilizar estrategias de RE, haciendo un uso desadaptativo de las mismas, y contribuyendo de este modo al mantenimiento de la sintomatología (Osma et al., 2021).

En los últimos años, la Terapia Cognitivo Conductual (TCC) ha propuesto intervenciones con un enfoque transdiagnóstico, es decir, centradas en los mecanismos subyacentes compartidos de los TEs (McManus et al., 2010). Como hemos mencionado en el párrafo anterior, este grupo de trastornos presenta problemas en la RE. A su vez, comparte una serie de mecanismos etiológicos y de mantenimiento, como el alto neuroticismo (Brown & Barlow, 2009). A este respecto, varios estudios sugieren que dimensiones elevadas de esta dimensión de personalidad se asocia directamente con diferentes trastornos médicos (Smith & MacKenzie, 2006; Suls et al., 2005), lo que indica que podría desempeñar un papel clave en la relación entre la CPCOVID y sus principales síntomas psicológicos.

El Protocolo Unificado para el Tratamiento Transdiagnóstico de los Trastornos Emocionales (PU; Barlow et al., 2018) es un ejemplo de intervención transdiagnóstica y cognitivo-conductual dirigida explícitamente al tratamiento del elevado neuroticismo, o lo que es lo mismo, al entrenamiento en habilidades de RE adaptativas (Barlow et al., 2018). Al centrarse en los mecanismos comunes en los distintos TE, el PU ofrece numerosas ventajas frente a los protocolos diseñados para el tratamiento de trastornos específicos, como el permitir el abordaje de personas que presentan comorbilidad (Brown et al., 2001).

Respecto a su utilidad, el PU ha demostrado eficacia en la reducción de síntomas de ansiedad y depresión, neuroticismo, síntomas específicos de cada trastorno y criterios diagnósticos y en el aumento de la extraversión, la calidad de vida y las habilidades de regulación emocional en personas con TEs, con y sin comorbilidad (Cassiello-Robbins et al., 2020; Leonardo et al., 2021; Sakiris y Berle, 2019). En España, nuestro equipo de investigación ha llevado a cabo el primer ensayo clínico multicéntrico aleatorizado aplicando el PU en formato grupal en pacientes con diagnóstico de TE en unidades de salud mental de nuestro Sistema Nacional de Salud (Osma et al., 2018), encontrando resultados similares (Osma, Peris-Baquero, Quilez-Orden, et al., 2021; Osma, Peris- Baquero, Suso-Ribera, et al., 2021). A su vez, una reciente revisión sistemática informa acerca de la utilidad preliminar del PU para el tratamiento de la sintomatología emocional en población que presenta además una condición médica, produciendo a su vez mejoras en la sintomatología médica en algunos casos (Osma et al. 2021).

Por todo lo mencionado anteriormente, y dado que la prevalencia de sintomatología emocional y TEs en personas con CPCOVID es alta, la aplicación del PU podría resultar de gran utilidad en el tratamiento de los mismos. De este modo,

nuestra hipótesis de partida plantea que una intervención psicológica estructurada centrada en tratar la disregulación emocional (el PU) generará una mejora en el estado emocional de este grupo de pacientes.

# OBJETIVOS DEL ESTUDIO

## Objetivo general:

Estudiar la eficacia y aceptabilidad del “Protocolo Unificado para el tratamiento transdiagnóstico de los trastornos emocionales” en el tratamiento de la sintomatología emocional y/o TEs en una muestra de pacientes con CPCOVID.

## Objetivos específicos:

1. Contribuir al avance del conocimiento sobre el tratamiento de la sintomatología emocional y/o TEs presentes en personas con CPCOVID.
2. Estudiar la eficacia del PU para el tratamiento de la sintomatología emocional y/o TEs a lo largo del tiempo a través de seguimientos al mes, y a los 3, 6 y 12 meses después de finalizar la intervención.
3. Evaluar la adherencia al programa, la aceptabilidad y la mejora en las medidas tras la aplicación del PU en formato individual online en una muestra de personas con CPCOVID.

# CONTRASTE DE HIPÓTESIS

## Hipótesis general:

El PU se mostrará eficaz para el tratamiento de la sintomatología emocional y/o TEs en una muestra de pacientes con CPCOVID.

## Hipótesis específicas:

H1. Se obtendrán mejoras estadísticamente significativas, con tamaños del efecto moderados, tras la intervención en todas las medidas utilizadas.

H2. Las mejoras obtenidas tras la aplicación del PU se mantendrán a medio y largo plazo, en los seguimientos al mes y a los 3, 6 y 12 meses.

H3. Los participantes reportarán puntuaciones de aceptabilidad y satisfacción elevadas respecto a la intervención, sus componentes y el formato de aplicación.

# METODOLOGÍA

## Población de estudio

La población que se incluirá en el estudio estará constituida por miembros de la cohorte constituida en el proyecto ARACoV 1 (aprobación CEICA acta Nº 14/2022) que no hayan sido incluidos en el ensayo “Intervención nutricional especializada y tratamiento rehabilitador para mejora de la calidad de vida en una cohorte de pacientes con condición post covid-19” perteneciente a dicho proyecto. Así, los participantes serán adultos, pertenecientes a la comunidad autónoma de Aragón, con criterios clínicos de haber padecido COVID-19 con diagnostico confirmado mediante técnicas y sistemas de diagnóstico (PCR, test rápidos de antígenos, test serológico de detección de anticuerpos) y que permanezcan con sintomatología más allá de 12 semanas tras el inicio de los síntomas.

## Criterios de selección

Los criterios de inclusión para la participación en el proyecto son: (1) Pertenecer a la comunidad autónoma de Aragón (2) Tener al menos 18 años, (3) Comprender bien el castellano (4) Infección por SARS CoV 2 documentada mediante PCR, test de Ag o serología (Ac anti N positivos) (5) Persistencia de los síntomas más allá de 12 semanas tras la infección aguda por SARS CoV 2, (6) Presentar una puntuación igual o superior a 8 puntos en sintomatología ansiosa (OASIS) y/o igual o superior a 7 puntos en sintomatología depresiva (ODSIS) (7) Disponer de acceso a Internet, (8) Firma del consentimiento informado.

Los criterios de exclusión son: (1) Participar en el ensayo “Intervención nutricional especializada y tratamiento rehabilitador para mejora de la calidad de vida en una cohorte de pacientes con condición post covid-19”, (2) Que el síntoma o síntomas ya existieran antes de la infección aguda por SARS CoV 2, (3) Estar

recibiendo tratamiento psicológico y/o farmacológico por un trastorno mental en el presente, (4) Tener un diagnóstico de trastorno mental grave, (5) Ideación suicida activa en el momento de la evaluación.

## Cálculo del tamaño de la muestra

Llevaremos a cabo un diseño experimental de caso único con línea de base múltiple para evitar la necesidad de un grupo control (en los diseños de caso la línea base se utiliza como control para cada participante). Las guías actuales (Kratochwill et al., 2010; Kratochwill et al., 2012) recomiendan 3 líneas bases distintas, así que los participantes se asignarán a una de estas 3 condiciones: inicio de la intervención tras 6 días de evaluación (condición 1), tras 8 días de evaluación (condición 2) o tras 10 días de evaluación (condición 3). Para calcular el tamaño de la muestra de este estudio piloto, nos basamos en un estudio que proponía reglas empíricas para los estudios piloto en función del tamaño del efecto deseado y del tamaño de un ensayo principal posterior (Bell et al., 2018). Así, para encontrar un tamaño del efecto de hasta 0.3, con una potencia del 80% y contando con que el estudio 2 tendrá una muestra de 130 participantes, el estudio de Bell et al. (2018) plantea que el tamaño muestral del estudio piloto debe ser de 20 participantes por condición, en este caso, al contar con tres líneas de base distintas, el tamaño muestral será de 60 participantes.

## Instrumentos

1. **Datos sociodemográficos e historia clínica:** sexo, edad, lugar de residencia, estado civil, situación laboral, hábitos de vida, vacunación SARS CoV 2.

## Protocolo de evaluación:

- - *Tabla de síntomas referidos por el paciente:* Para facilitar el recogimiento de los síntomas referidos por el paciente respecto a la CPCOVID, hemos desarrollado una tabla en la que se recogen distintos síntomas presentes en esta condición, agrupados en las siguientes categorías: Síntomas Generales, Síntomas Respiratorios, Síntomas Gastrointestinales, Síntomas Musculoesqueléticos, Síntomas Cutáneos, Síntomas Otorrinolaringológicos, Síntomas Neurológicos, Síntomas Cardiovasculares y Otros.
  - *Entrevista estructurada para los trastornos de ansiedad y trastornos relacionados, según el DSM-5 (ADIS-5; Brown y Barlow, 2014):* Entrevista estructurada que sigue los criterios DSM-5 para los trastornos de ansiedad, estado de ánimo y relacionados. Se evaluarán los trastornos adaptativos siguiendo los criterios DSM-5.
  - *Escala General de Gravedad e Interferencia de la Depresión (ODSIS; Bentley et al., 2014; Osma et al., 2019):* Evaluación a través de 5 ítems de la frecuencia, intensidad, gravedad e interferencia de la sintomatología depresiva.
  - *Escala General de Gravedad e Interferencia de la Ansiedad (OASIS; Norman et al., 2006; Osma et al., 2019):* Evaluación a través de 5 ítems de la frecuencia, intensidad, gravedad e interferencia de la sintomatología ansiosa.
  - *EuroQol-5D (Brooks, 1996. Validada al castellano por Badia et al., 1999):*

Evaluación del estado de salud autopercibido.

- - *Inventario Multidimensional para los Trastornos Emocionales (MEDI; Rosellini y Brown, 2019; Osma et al., 2022):* Evaluación a través de 49 ítems del perfil transdiagnóstico de los Trastornos Emocionales, el cual se compone de nueve dimensiones: temperamento neurótico, temperamento positivo, estado de ánimo deprimido, ansiedad somática, activación del arousal, ansiedad social, cogniciones intrusivas, reexperimentación traumática, y evitación.
  - *Escala de Dificultades en la Regulación emocional (DERS; Gratz y Roemer, 2004. Validada al castellano por Hervás y Jódar, 2008):* Evaluación a través de 28 ítems de las dificultades en regulación emocional por medio de 5 subescalas: descontrol, rechazo, interferencia, desatención y confusión emocional.
  - *Escala de tolerancia al estrés (DTS; Simons y Gaher, 2005. Validada al castellano por Sandin et al., 2017):* Evaluación a través de 15 ítems de la tolerancia al malestar. Evalúa las siguientes dimensiones: 1) Capacidad percibida para tolerar el malestar emocional; 2) Valoración subjetiva del malestar; 3) Atención absorbida por las emociones negativas; 4) Esfuerzos de regulación para aliviar el malestar.
  - *Cuestionario de Satisfacción con el Tratamiento (STQ; adaptación del Client Satisfaction Questionnaire [CSQ-8] de Larsen et al., 1979):* Nuestra adaptación incluye 6 de los 8 ítems del CSQ-8 (calidad percibida, adecuación a las expectativas previas, recomendación del tratamiento a amigos o familiares, utilidad de las técnicas aprendidas, satisfacción general con la intervención y probabilidad de que vuelvan a elegir una intervención de este tipo) y un ítem más relativo al malestar que ha generado la intervención. Asimismo, se ha realizado un cambio en la escala Likert de respuesta pasando de 4 puntos en la original (0= “Mal / Nada” a 4= “Excelente/Mucho”) a 11 en la actual (0= “Mal / Nada a 10= "Excelente/Mucho"). Además, se añadieron las siguientes 5 preguntas en formato abierto: *¿Hay algún otro contenido que crees que sería interesante incluir en el programa?; ¿Crees que hay algún contenido del programa que no es necesario abordar?; La duración del programa, 5 sesiones de 2 horas, ¿crees que es suficiente?; ¿Cuál es tu opinión acerca del tipo de formato utilizado para aplicar el programa?; Te dejamos a continuación un espacio para que puedas expresar cualquier cuestión acerca del programa.*
  - *Cuestionario de evaluación de los módulos del Protocolo Unificado (PU):* Cuestionario elaborado *ad hoc* compuesto por 7 preguntas; una de carácter general que evalúa la utilidad del programa para mejorar la regulación emocional y seis específicas que evalúan por separado la utilidad para regular mejor las emociones de cada una de las técnicas que se trabajan en los diferentes módulos del PU. La escala de respuesta es tipo Likert y va de 0 (nada) a 10 (muchísimo).
  - *Cuestionario de evaluación de la línea base: P*ara la evaluación diaria de la línea base, utilizaremos los cuestionarios ODSIS (Bentley et al., 2014; Osma et al., 2019) y OASIS (Norman et al., 2006; Osma et al., 2019) modificando la temporalidad a la que hacen referencia las preguntas; pasando de “durante la última semana” en los cuestionarios originales a “durante el día de ayer” en la versión utilizada para la evaluación de la línea base.

## Análisis estadístico

Los análisis serán llevados a cabo empleando el paquete estadístico IBM SPSS Statistics version 22.0 para Windows (Corp. IBM, 2013). En primer lugar, se llevarán a cabo pruebas de normalidad para comprobar si la muestra sigue o no una distribución normal. A continuación, se llevarán a cabo análisis estadísticos descriptivos con el objetivo de obtener una visión general de las puntuaciones en las variables y datos sociodemográficos. Se llevarán a cabo análisis paramétricos o no paramétricos (en función de si la muestra sigue o no una distribución normal). En primer lugar, se realizarán análisis de varianza (ANOVA) con el objetivo de ver si hay diferencias entre las tres condiciones en los distintos momentos de evaluación. En segundo lugar, se llevarán a cabo análisis ANOVA de medidas repetidas para analizar la evolución en cada una de las condiciones. En el caso de que la muestra no siga una distribución normal, se llevarán a cabo pruebas no paramétricas equivalentes con el mismo objetivo. Para todos los análisis estadísticos, se calcularán los tamaños del efecto a través del estadístico d de Cohen, cuyas estimaciones suelen interpretarse como pequeñas (d ≈ 0,2), medianas (d ≈ 0,5) o grandes (d ≈ 0,8). Otro aspecto que consideramos importante analizar es cómo los participantes cambian sus puntuaciones en las escalas ODSIS y OASIS en función del contenido abordado en cada módulo. Para ello se realizará un análisis visual de los cambios en las puntuaciones a través del software estadístico R (versión 4.1.0; R Core Team, 2021), para ver cómo cambian las pendientes en las distintas fases del estudio (evaluación y tratamiento), y en los distintos módulos dentro del tratamiento. Para llevar a cabo este análisis visual, se utilizarán las respuestas de las escalas ODSIS y OASIS que los participantes rellenarán en los momentos pre- programa, semanalmente durante el programa, post-programa y en los seguimientos al mes y 3 meses. Por último, se llevarán a cabo análisis cualitativos, concretamente análisis de contenido, mediante el software estadístico MAXQDA (Kuckartz y Rädiker, 2019) para analizar las respuestas a las preguntas abiertas sobre la evaluación de la satisfacción del programa, sus contenidos y el formato.

## Intervenciones a realizar

- - 1. **Reclutamiento y difusión del estudio**

El reclutamiento de los participantes se realizará a través de la cohorte constituida en el proyecto ARACoV 1 (aprobación CEICA acta Nº 14/2022). Desde el proyecto ARACoV 1, se informará acerca del estudio y se le ofrecerá la posibilidad de participar a aquellas personas pertenecientes a la cohorte ARACoV 1 que no hayan sido incluidos en el ensayo “Intervención nutricional especializada y tratamiento rehabilitador para mejora de la calidad de vida en una cohorte de pacientes con condición post covid-19” perteneciente a dicho proyecto y que presenten una

puntuación igual o superior a 8 puntos en sintomatología ansiosa (OASIS) y/o igual o superior a 7 puntos en sintomatología depresiva (ODSIS), habiendo cumplimentado estas escalas dentro del proyecto ARACoV 1 y de manera posterior a la firma del consentimiento informado de dicho proyecto. Aquellas personas interesadas en participar recibirán la hoja de información sobre el estudio y consentimiento informado por parte del enfermero/a miembro del equipo del proyecto ARACoV 1 encargado del reclutamiento de la cohorte ARACoV 1 que previamente le ha informado acerca del estudio. Tras haber accedido a toda la información sobre el estudio recogida en la hoja de información y consentimiento informado, aquellos que deseen participar, podrán firmar el consentimiento informado sobre el mismo y la LOPD.

Con aquellos que acepten participar, se concertará una sesión online individual con la psicóloga encargada de llevar a cabo la intervención en la que se llevará a cabo una evaluación de cara a conocer el posible trastorno emocional del paciente, así como descartar la presencia de un trastorno mental grave y/o ideación suicida activa, siendo estos dos últimos criterios de exclusión. Si tras la evaluación se detectase un posible problema de salud mental cuya gravedad requiera de intervención especializada y diferente a la proporcionada mediante el presente programa; se informará al paciente de que no puede continuar con su participación en el estudio al tratarse de un criterio de exclusión y se recomendará al participante que busque ayuda profesional y se le orientará para ello. Tras la evaluación, se mandará al participante un enlace de Google Forms a través del cual pasará a responder el protocolo de evaluación pre-intervención.

Una vez lo hayan completado, se les informará acerca de que en las próximas semanas recibirán un email en el que se les notificará acerca de la condición a la que han sido asignados aleatoriamente: inicio de la intervención tras 6 días de evaluación (condición 1; 6 días de línea base), tras 8 días de evaluación (condición 2; 8 días de línea base) o tras 10 días de evaluación (condición 3; 10 días de línea base). La aleatorización a las diferentes líneas de base se realizará con el software de aleatorización ([www.randomizer.org).](http://www.randomizer.org/)

## Desarrollo de la intervención

La idea fundamental de elegir este tipo de diseño es que cada uno de los participantes puede ser su propio control (Kratochwill et al., 2013). Así, como se ha explicado con anterioridad, los participantes serán aleatorizados a una de las 3 condiciones, 6, 8 o 10 días de línea de base. Cada condición estará compuesta por 20 participantes.

El programa de intervención basado en el PU se llevará a cabo durante 8 sesiones de 1 hora de duración, en formato online mediante la plataforma Google Meet. Durante las sesiones se corregirán los ejercicios de la sesión anterior (salvo en la sesión 1), se introducirán los nuevos contenidos de la sesión, se hará una encuesta de V/F para asegurar que han comprendido los conceptos clave de la sesión, se realizarán ejercicios en sesión y se animará a la práctica de los ejercicios entre sesiones.

Los/Las participantes recibirán un manual breve donde aparecerán los contenidos importantes de cada sesión resumidos, los ejercicios a realizar y los registros correspondientes. Los contenidos de las sesiones serán:

| **Título** | **Contenido / Habilidad de Regulación emocional** | **Práctica** |
| --- | --- | --- |
| Sesión 1. Establecimiento de objetivos y motivación para el cambio | Establecimiento de objetivos de tratamiento  Ejercicio de Balance decisional | Definir los objetivos generales de tratamiento, así como los pasos para alcanzarlos. Explorar los pros y contras de cambiar y de permanecer igual, reflexionando sobre ello para inclinar la balanza a favor del cambio. |
| Sesión 2. Comprendiendo tus emociones | Análisis funcional de la respuesta emocional | Analizar la respuesta emocional a través del registro ARCO y valorar las consecuencias a corto y largo  plazo de nuestras respuestas emocionales. |
| Sesión 3. Conciencia emocional plena | Observar lo que ocurre en el presente sin juzgar | Práctica de distintos ejercicios de meditación |
| Sesión 4. Flexibilidad Cognitiva | Observar nuestros pensamientos y abrir el abanico de posibilidades de interpretación | Observar los pensamientos que tenemos y averiguar si reflejan la realidad y nos acercan a nuestros objetivos y, si no es así, entrenar en |

|  | Técnica de solución de Problemas | generar nuevas interpretaciones de la situación que sean más  realistas y útiles/o aplicar solución de problemas. |
| --- | --- | --- |
| Sesión 5. Oponiéndose a las conductas emocionales | Identificar las conductas emocionales que realizamos y describir conductas opuestas o alternativas | Identificaremos las conductas emocionales que utilizamos, valoraremos su utilidad, describiremos conductas alternativas u opuestas y valoraremos su utilidad. |
| Sesión 6. Exposiciones emocionales I | Exposición gradual a los estímulos internos y/o externos que nos generan malestar intenso para tolerar el malestar progresivamente | Diseñaremos y realizaremos ejercicios que provoquen las sensaciones físicas presentes en las emociones y las toleraremos poco apoco hasta que ya no tengan un efecto negativo en nuestra conducta. |
| Sesión 7. Exposiciones emocionales II | Exposición gradual a los estímulos internos y/o externos que nos generan malestar intenso para tolerar el malestar progresivamente | Elaboraremos una jerarquía de exposición a distintas emociones para ir exponiéndonos progresivamente a cada una de ellas y así tolerarlas poco a poco hasta que ya no tengan un efecto negativo en nuestra  conducta. |
| Sesión 8. Prevención de recaídas | Repaso de las habilidades aprendidas y del progreso durante la intervención.  Plan de práctica tras la intervención.  Registro de situaciones difíciles y modo de afrontarlas. | Repasaremos el progreso para tener una visión general de todo lo aprendido y ser consciente de las habilidades que necesitamos seguir entrenando.  Identificaremos las situaciones difíciles de riesgo para poder reconocerlas a tiempo y así saber qué habilidades tenemos para hacerles frente y asegurarnos de haberlas practicado. |

Una vez finalizada la intervención se les enviará un enlace de Google Forms por email para rellenar el protocolo post-programa. Se realizarán seguimientos tras la finalización del programa al mes y a los 3, 6 y 12 meses siguiendo el mismo procedimiento del post-programa.

# ASPECTOS ÉTICOS

Todas las personas que sean evaluadas y cumplan los criterios de inclusión para participar en el ensayo firmarán el documento de Protección de Datos de Carácter Personal para que tengan noción de quién va a utilizar los resultados de esta investigación y con qué fin.

Los participantes que vayan a recibir intervención serán informados sobre qué consiste el tratamiento, al igual que su duración y las fases del estudio. Para ello se les hará llegar una hoja de información explicando el tratamiento, y sus respectivas fases, así como el documento correspondiente al Consentimiento Informado.

En cuanto a la confidencialidad, el tratamiento, la comunicación y la cesión de los datos de carácter personal de todos los sujetos participantes, se ajustará a lo dispuesto en la Declaración de Helsinki (Seúl, 2008), Ley 14/2007 de Investigación biomédica, Ley Orgánica 3/2018, de 5 de diciembre, de Protección de Datos Personales y de Garantía de Derechos Digitales (LOPD GDD). A partir del 25 de mayo de 2018 es de plena aplicación la nueva legislación en la UE sobre datos personales, en concreto el Reglamento (UE) 2016/679 del Parlamento europeo y del Consejo de 27 de abril de 2016 de Protección de Datos (RGPD). De acuerdo a lo que establece la legislación mencionada, los participantes pueden ejercer los derechos de acceso, modificación, oposición y cancelación de datos, para lo cual deberá dirigirse al investigador principal a cargo del estudio. La información personal recogida para el estudio será sustituida por códigos alfanuméricos y los datos sociodemográficos se guardarán de manera separada y solo se tendrá acceso por parte de los investigadores responsables del almacenamiento y tratamiento de los datos, siempre protegiendo el derecho a la privacidad.

La información se recogerá mediante la aplicación Google Forms, la cual se incluye dentro del paquete "Google Workspace for Education" de Unizar. A través de este paquete, google ofrece correo electrónico, documentos y espacio de almacenamiento a la Universidad de Zaragoza. Este paquete de programas es ofrecido en modalidad nube y, por tanto, deben tenerse en cuenta las características y vinculación en relación con la normativa aplicable en España, que a considerar de forma inicial serían el Esquema Nacional de Seguridad y la norma vigente en materia de Protección de Datos y Garantía de Derechos Digitales. En este sentido, tal y como señala el propio fabricante, las soluciones Cloud de Google han obtenido la conformidad para el Esquema Nacional de Seguridad “Nivel ALTO”.

Por otro lado, y atendiendo a la normativa para la protección de Datos de Carácter Personal, existe el compromiso y declaración por parte de Google para su cumplimiento. Asimismo, los servicios ofrecidos por la empresa Google se circunscriben a los requisitos regulados a través del Reglamento Europeo 2016/679 (GDPR), dentro del acuerdo en materia de protección de datos de países terceros con la Unión Europea. Google Workspace for Education se rige por las siguientes normas y certificaciones:

ISO / IEC 27001 (Gestión de la seguridad de la información) ISO / IEC 27017 (seguridad en la nube)

ISO / IEC 27018 (Privacidad en la nube)

ISO / IEC 27701 (Gestión de la información de privacidad)

# CRONOGRAMA

El reclutamiento de los participantes está previsto que comience en octubre 2022 y se prolongue hasta junio 2023.

# PRESUPUESTO

- Contrato de técnico superior de investigación a tiempo completo para realizar las intervenciones (inicio contrato 3 octubre 2022 hasta 3 octubre 2023). El contrato corre a cargo del proyecto ARACoV 1 gestionado por el Instituto de Investigación Sanitaria de Aragón.

# BIBLIOGRAFÍA

Barlow, D. H., Farchione, T. J., Sauer-Zavala, S., Latin, H. M., Ellard, K. K., Bullis, J. R., Bentley, K., Boettcher, H., y Cassiello-Robbins, C. (2018). *Unified protocol for transdiagnostic treatment of emotional disorders: Therapist guide (2nd ed.).* New York, NY: Oxford University Press.

Bell, M. L., Whitehead, A. L., & Julious, S. A. (2018). Guidance for using pilot studies to inform the design of intervention trials with continuous outcomes. *Clinical epidemiology, 10,* 153–157. https://doi.org/10.2147/CLEP.S146397

Bentley, K., Gallagher, M., y Barlow, D. (2014). Development and validation of the Overall Depression Severity and Impairment Scale. *Psychological Assessment*, *26*(3), 815–830. https://doi.org/10.1037/a0036216

Brown, T. A., y Barlow, D. H. (2009). A proposal for a dimensional classification system based on the shared features of the DSM-IV anxiety and mood disorders: Implications for assessment and treatment. *Psychological Assessment*, *21*(3), 256. <https://doi.org/10.1037/a0016608>

Brown, T. A., & Barlow, D. H. (2014). Anxiety and related disorders interview schedule for DSM-5 (ADIS-5)-adult and lifetime version: Clinician manual. Oxford University Press.

Brown, T. A., Campbell, L. A., Lehman, C. L., Grisham, J. R., y Mancill, R. B. (2001). Current and lifetime comorbidity of the DSM-IV anxiety and mood disorders in a large clinical sample. *Journal of Abnormal Psychology*, *110*(4), 585. [https://doi.org/10.1037//0021-843x.110.4.585](https://doi.org/10.1037/0021-843x.110.4.585)

Brown, D. A., & O’Brien, K. K. (2021). Conceptualising Long COVID as an episodic health condition. *BMJ Global Health*, *6*(9), e007004.

Cassiello-Robbins, C., Southward, M. W., Tirpak, J. W., y Sauer-Zavala, S. (2020). A systematic review of Unifed Protocol applications with adult populations:

Facilitating widespread dissemination via adaptability. *Clinical Psychology Review, 78,* 101852.

Fernández-de-Las-Peñas, C., Palacios-Ceña, D., Gómez-Mayordomo, V., Cuadrado, M. L., & Florencio, L. L. (2021). Defining post-COVID symptoms (post-acute COVID, long COVID, persistent post-COVID): an integrative classification. *International journal of environmental research and public health*, *18*(5), 2621.

Gobierno de Aragón (2022). Covid 19. Situación actual. <https://transparencia.aragon.es/COVID19>

Gratz, K. L. (2003). Risk factors for and functions of deliberate self-harm: An empirical and conceptual review. *Clinical Psychology: Science and Practice*, *10*(2), 192.

Gratz, K. L., y Roemer, L. (2004). Multidimensional assessment of emotion regulation and dysregulation: Development, factor structure, and initial validation of the difficulties in emotion regulation scale. *Journal of psychopathology and behavioral assessment*, *26*(1), 41-54.

Gross, J. J. (1999). Emotion regulation: Past, present, future. *Cognition & emotion*, *13*(5), 551-573.

Hervás, G., y Jódar, R. (2008). Adaptación al castellano de la Escala de Dificultades en la Regulación Emocional. *Clínica y Salud*, *19*(2), 139–156.

Khraisat, B., Toubasi, A., AlZoubi, L., Al-Sayegh, T., & Mansour, A. (2021). Meta- analysis of prevalence: the psychological sequelae among COVID-19 survivors. *International Journal of Psychiatry in Clinical Practice*, 1-10

Kratochwill, T. R., Hitchcock, J., Horner, R. H., Levin, J. R., Odom, S. L., Rindskopf,

D. M., & Shadish, W. R. (2010). Single-case designs technical documentation. What works clearinghouse.

Kratochwill, T. R., Hitchcock, J. H., Horner, R. H., Levin, J. R., Odom, S. L., Rindskopf, D. M., & Shadish, W. R. (2013). Single-case intervention research design standards. *Remedial and Special Education*, *34*(1), 26-38.

Larsen, D. L., Atkinson, C. C., Hargreaves, W. A., y Nguyen, T. D. (1979). Assessment of client/patient satisfaction: Development of a general scale. *Evaluation and Program Planning, 2*, 197-207.

Leonardo, C., Aristide, S., y Michela, B. (2021). On the efficacy of the Barlow Unified Protocol for Transdiagnostic Treatment of Emotional Disorders: A systematic review and meta-analysis. *Clinical Psychology Review*, 101999.

Leviner, S. (2021). Recognizing the clinical sequelae of COVID-19 in adults: COVID- 19 Long-Haulers. *The Journal for Nurse Practitioners*, *17*(8), 946-949.

Linehan, M. M. (1993). *Cognitive-behavioral treatment of borderline personality disorder.* Guilford Press.

Mandal, S., Barnett, J., Brill, S. E., Brown, J. S., Denneny, E. K., Hare, S. S., ... & Hurst, J. R. (2021). ‘Long-COVID’: a cross-sectional study of persisting symptoms, biomarker and imaging abnormalities following hospitalisation for COVID-19. *Thorax, 76*(4), 396-398.

Mazza, M. G., De Lorenzo, R., Conte, C., Poletti, S., Vai, B., Bollettini, I., Melloni, E., Furlan, R., Ciceri, F., Rovere-Querini, P., COVID-19 BioB Outpatient Clinic Study group, & Benedetti, F. (2020). Anxiety and depression in COVID-19 survivors: Role of inflammatory and clinical predictors. *Brain, behavior, and immunity*, *89*, 594–600. <https://doi.org/10.1016/j.bbi.2020.07.037>

McManus, F., Shafran, R., y Cooper, Z. (2010). What does a transdiagnostic approach have to offer the treatment of anxiety disorders? *British Journal of Clinical Psychology, 49*(4), 491–505. <https://doi.org/10.1348/014466509X476567>

Norman, S. B., Hami Cissell, S., Means-Christensen, A. J., y Stein, M. B. (2006). Development and validation of an Overall Anxiety Severity And Impairment Scale (OASIS). *Depression and Anxiety*, *23*(4), 245–249. https://doi.org/10.1002/da.20182

Office for National Statistics. (2021). Coronavirus and the social impacts of ‘long COVID’on people’s lives in Great Britain: 7 April to 13 June 2021.

Osma, J., Martínez-Loredo, V., Quilez-Orden, A., Peris-Baquero, Ó., & Suso-Ribera, C. (2021). Validity Evidence of the Multidimensional Emotional Disorders Inventory among Non-Clinical Spanish University Students. *International Journal of Environmental Research and Public Health*, *18*(16), 8251.

Osma, J., Martínez-García, L., Quilez-Orden, A., & Peris-Baquero, Ó. (2021). Unified Protocol for the Transdiagnostic Treatment of Emotional Disorders in Medical Conditions: A Systematic Review. *International journal of environmental research and public health*, *18*(10), 5077.

Osma, J., Peris-Baquero, Ó., Quilez-Orden, A., Suso-Ribera, C. y Crespo, E. (2021). Protocolo Unificado para el Tratamiento Transdiagnóstico de los Trastornos Emocionales. En E. Fonseca (Ed.), *Manual de Tratamientos Psicológicos: Adultos* (pp. 195-220). Madrid: Ediciones Pirámide.

Osma, J., Peris-Baquero, O., Suso-Ribera, C., Farchione, T. J., y Barlow, D. H. (2021). Effectiveness of the Unified Protocol for transdiagnostic treatment of emotional disorders in group format in Spain: Results from a randomized controlled trial with 6-months follow-up. *Psychotherapy Research*, 1–14.

Osma, J., Quilez-Orden, A., Suso-Ribera, C., Peris-Baquero, O., Norman, S., Bentley, K., y Sauer-Zavala, S. (2019). Psychometric properties and validation of the Spanish versions of the overall anxiety and depression severity and impairment scales. *Journal of Affective Disorders*, *252*, 9–18. https://doi.org/10.1016/j.jad.2019.03.063

Osma, J., Suso-Ribera, C., Garcia-Palacios, A., Crespo-Delgado, E., Robert-Flor, C., Sanchez-Guerrero, A., ... Torres-Alfosea, M. Á. (2018). Efficacy of the unified protocol for the treatment of emotional disorders in the Spanish public mental health system using a group format: study protocol for a multicenter, randomized, non-inferiority controlled trial. *Health and quality of life outcomes, 16*(1), 1-10.

Rosellini, A. J., y Brown, T. A. (2019). The Multidimensional Emotional Disorder Inventory (MEDI): Assessing transdiagnostic dimensions to validate a profile approach to emotional disorder classification. *Psychological assessment*, *31*(1), 59.

Sakiris, N., y Berle, D. (2019). A systematic review and meta-analysis of the Unified Protocol as a transdiagnostic emotion regulation based intervention. *Clinical psychology review, 72*, 101751. <https://doi.org/10.1016/j.cpr.2019.101751>

Sandín Ferrero, B., Simons, J. S., Valiente García, R. M., Simons, R. M., & Chorot Raso, P. (2017). Psychometric properties of the spanish version of The Distress Tolerance Scale and its relationship with personality and psychopathological symptoms. *Psicothema*.

Simons, J. S., & Gaher, R. M. (2005). The Distress Tolerance Scale: Development and validation of a self-report measure. *Motivation and emotion*, *29*(2), 83-102.

Smith, T. W., y MacKenzie, J. (2006). Personality and risk of physical illness. *Annual Review of Clinical Psychology, 2*, 435–467. <https://doi.org/10.1146/annurev.clinpsy.2.022305.095257>

Suls, J., y Bunde, J. (2005). Anger, anxiety, and depression as risk factors for cardiovascular disease: the problems and implications of overlapping affective dispositions. *Psychological bulletin*, *131*(2), 260–300. [https://doi.org/10.1037/0033-](https://doi.org/10.1037/0033-2909.131.2.260)

[2909.131.2.260](https://doi.org/10.1037/0033-2909.131.2.260)

Thye, A. Y. K., Law, J. W. F., Tan, L. T. H., Pusparajah, P., Ser, H. L., Thurairajasingam, S., ... & Lee, L. H. (2022). Psychological Symptoms in COVID-19 Patients: Insights into Pathophysiology and Risk Factors of Long COVID-

19. *Biology*, *11*(1), 61.

Vilagut, G., Valderas, J. M., Ferrer, M., Garin, O., López-García, E., & Alonso, J. (2008). Interpretación de los cuestionarios de salud SF-36 y SF-12 en España: componentes físico y mental. *Medicina clínica*, *130*(19), 726-735.

Ware Jr, J. E., Kosinski, M., & Keller, S. D. (1996). A 12-Item Short-Form Health Survey: construction of scales and preliminary tests of reliability and validity. *Medical care*, 220-233.

World Health Organization. (2021). A clinical case definition of post COVID-19 condition by a Delphi consensus, 6 October 2021.

[https://www.who.int/publications/i/item/WHO-2019-nCoV-Post_COVID-](https://www.who.int/publications/i/item/WHO-2019-nCoV-Post_COVID-19_conditionClinical_case_definition-2021.1) [19_conditionClinical_case_definition-2021.1](https://www.who.int/publications/i/item/WHO-2019-nCoV-Post_COVID-19_conditionClinical_case_definition-2021.1)

World Health Organization. (2022). WHO Coronavirus disease (COVID‐19) dashboard.
